# Supplementary material for: Formative peer assessment in higher healthcare education programmes: a scoping review
Source: BMJ Open. 2021 Feb 8;11(2):e045345. doi: 10.1136/bmjopen-2020-045345 (PMC7875268; doi:10.1136/bmjopen-2020-045345)
Supplement: Supplementary data [file bmjopen-2020-045345supp001.pdf]

Appendix 1. Presentation of the search strategy and results from the Pub Med database until 28th of May, 2019.

	Search block 1: Healthcare education
#	MESH-terms
1	"Students, Medical"[Mesh] OR
2	"Students, Nursing"[Mesh] OR
3	"Students, Dental"[Mesh] OR
4	"Students, Health Occupations"[Mesh] OR
5	"Education, Medical"[Mesh] OR
6	"Education, Nursing"[Mesh] OR
7	"Education, Dental" [Mesh] OR
8	"Midwifery/education"[Mesh] OR
9	"Allied Health Personnel/education"[Mesh]
10	S1 OR S2 OR S3 OR S4 OR S5 OR S6 OR S7 OR S8 OR S9
#	Free text terms
11	"medical student*" OR
12	"nursing student*" OR
13	"midwifery student* OR
14	"dental student*" OR
15	"physical therapy student*" OR
16	"occupational therapy student*" OR
17	"allied health student*" OR
18	"health occupations student*" OR
19	"health care stud*" OR
20	"Health care education" OR
21	"health science education" OR
22	"Medical education" OR
23	" Nursing education" OR
24	"Dental education" OR
25	"allied health education" OR
26	"Health occupation* education*" OR
27	"midwifery education"
28	S11 OR S12 OR S13 OR S14 ORS15 OR S16 OR S17 OR S18 OR S19 OR S20 OR S21 OR S22 OR S23 OR S24 OR S25 OR S26 OR S27
29	S10 OR S28

	Search block 2: Peer assessment
#	MESH
31	"Educational Measurement"[Mesh] OR
32	"Peer Group"[Mesh] OR
33	"Peer Review"[Mesh]
34	S31 OR S32 OR S33
#	Free text terms
35	"peer assessment" OR
36	"peer evaluation" OR
37	"peer observation" OR
38	"peer feedback" OR
39	"peer review" OR
40	"peer assess*" OR
41	"*peer assess" OR
42	"peer examiner" OR
43	"peer grad*" OR
44	"peer group" OR
45	"Student performance appraisal" OR
46	"educational measurement"
47	S35 OR S36 OR S37 OR S38 OR S39 OR S40 OR S41 OR S42 OR S43 OR S44 OR S45 OR S46
48	S34 OR S47

	Search block 3: Formative assessment
#	MESH
49	"Formative Feedback"[Mesh]
#	Free text terms
50<br>51<br>52<br>53<br>54<br>55<br>56<br><br>57	"Formative evaluation" OR<br>"Formative feedback" OR<br>“Formative assessment” OR<br>“Formative* assess*” OR<br>“Formativ* evaluation” OR<br>Formativ* OR<br>“formative evaluation research”<br><br>S37 OR S38 OR S39 OR S40 OR S41 OR S42 OR S43
58	S49 OR S57

#	Combination of search blocks 1, 2, and 3
59	S29 AND S48 AND S58
