# Supplementary material for: Formative peer assessment in higher healthcare education programmes: a scoping review
Source: BMJ Open. 2021 Feb 8;11(2):e045345. doi: 10.1136/bmjopen-2020-045345 (PMC7875268; doi:10.1136/bmjopen-2020-045345)
Supplement: Supplementary data [file bmjopen-2020-045345supp002.pdf]

Appendix 2. Charting form

CHARTING FORM

ARTICLE NO:

TITLE:

AUTHOR/S:

YEAR OF PUBLICATION:

COUNTRY:

RESEARCH DESIGN: QUANTITATIVE ☐ QUALITATIVE ☐ OTHER: ☐ ________

METHOD:

AIM:

NUMBER OF PARTICIPANTS (n=):

SETTING:

MEDICAL EDUCATION ☐

NURSING EDUCATION ☐

MIDWIFERY EDUCATION ☐

PHYSIOTHERAPY EDUCATION ☐

OCCUPATIONAL THERAPY ☐

DENTAL EDUCATION ☐

OTHER ☐ ________

DOES THE STUDY PRESENT?

HOW PEER ASSESSEMENT INTERVENTIONS ARE DELIVERED	YES	NO	
PEER ASSESSMENT	YES	NO	
FORMATIVE ASSESSMENT	YES	NO	
STUDENTS EXPERIENCE	YES	NO	
TEACHERS EXPERIENCE	YES	NO	OTHER:
OUTCOME/S OF PEER ASSESSMENT INTERVENTION	YES	NO	
RATIONALE/S FOR PEER ASSESSMENT INTERVENTION	YES	NO	

MAIN FINDINGS:

INCLUDED: YES NO

REASON/S FOR EXCLUSION:

(Only to be answered if the study is to be included)

Grading according to the Critical Appraisal Skills Program (CASP)

QUALITATIVE STUDIES AND SYSTEMATIC REVIEWS

LOW ☐ MODERATE ☐ HIGH ☐

RCT AND CASE CONTROL STUDIES

LOW ☐ MODERATE ☐ HIGH ☐

COHORT STUDIES

LOW ☐ MODERATE ☐ HIGH ☐

Grading according to The Mixed Method Appraisal Tool

MIXED METHOD STUDIES

LOW ☐ MODERATE ☐ HIGH ☐

Grading according to the Joanna Briggs Institute Critical Appraisal Tool

QUASI-EXPERIMENTAL STUDIES

LOW ☐ MODERATE ☐ HIGH ☐

ANALYTICAL CROSS SECTIONAL STUDIES

LOW ☐ MODERATE ☐ HIGH ☐

CASE REPORTS

LOW ☐ MODERATE ☐ HIGH ☐

Reviewed by:

(Signature and date)
