# Supplementary material for: Formative peer assessment in higher healthcare education programmes: a scoping review
Source: BMJ Open. 2021 Feb 8;11(2):e045345. doi: 10.1136/bmjopen-2020-045345 (PMC7875268; doi:10.1136/bmjopen-2020-045345)
Supplement: Author's manuscript [file bmjopen-2020-045345.draft_revisions.pdf]

BMJ Open is committed to open peer review. As part of this commitment we make the peer review history of every article we publish publicly available.

When an article is published we post the peer reviewers' comments and the authors' responses online. We also post the versions of the paper that were used during peer review. These are the versions that the peer review comments apply to.

The versions of the paper that follow are the versions that were submitted during the peer review process. They are not the versions of record or the final published versions. They should not be cited or distributed as the published version of this manuscript.

BMJ Open is an open access journal and the full, final, typeset and author-corrected version of record of the manuscript is available on our site with no access controls, subscription charges or pay-per-view fees (http://bmjopen.bmj.com).

If you have any questions on BMJ Open's open peer review process please email info.bmjopen@bmj.com

Formative Peer Assessment in Higher Healthcare Education Programs – a Systematic Scoping Review

Journal:	*BMJ Open*
Manuscript ID	bmjopen-2020-045345
Article Type:	Original research
Date Submitted by the Author:	03-Oct-2020

Complete List of Authors:	Stenberg, Marie; Malmö Universitet, Care Science<br>Mangrio, Elisabeth; Malmö Universitet, Care Science; Malmö Universitet, Malmö Institute for Studies of Migration, Diversity and Welfare<br>Bengtsson, Mariette; Malmö Universitet, Care Science<br>Carlson, Elisabeth; Malmö Universitet, Care Science

Keywords:	MEDICAL EDUCATION & TRAINING, EDUCATION & TRAINING (see Medical Education & Training), Quality in health care < HEALTH SERVICES ADMINISTRATION & MANAGEMENT

SCHOLARONE™
Manuscripts

I, the Submitting Author has the right to grant and does grant on behalf of all authors of the Work (as defined in the below author licence), an exclusive licence and/or a non-exclusive licence for contributions from authors who are: i) UK Crown employees; ii) where BMJ has agreed a CC-BY licence shall apply, and/or iii) in accordance with the terms applicable for US Federal Government officers or employees acting as part of their official duties; on a worldwide, perpetual, irrevocable, royalty-free basis to BMJ Publishing Group Ltd ("BMJ") its licensees and where the relevant Journal is co-owned by BMJ to the co-owners of the Journal, to publish the Work in this journal and any other BMJ products and to exploit all rights, as set out in our licence.

The Submitting Author accepts and understands that any supply made under these terms is made by BMJ to the Submitting Author unless you are acting as an employee on behalf of your employer or a postgraduate student of an affiliated institution which is paying any applicable article publishing charge ("APC") for Open Access articles. Where the Submitting Author wishes to make the Work available on an Open Access basis (and intends to pay the relevant APC), the terms of reuse of such Open Access shall be governed by a Creative Commons licence – details of these licences and which Creative Commons licence will apply to this Work are set out in our licence referred to above.

Other than as permitted in any relevant BMJ Author's Self Archiving Policies, I confirm this Work has not been accepted for publication elsewhere, is not being considered for publication elsewhere and does not duplicate material already published. I confirm all authors consent to publication of this Work and authorise the granting of this licence.

For peer review only

Formative Peer Assessment in Higher Healthcare Education Programs – a Systematic Scoping Review

Marie Stenberg[1] RN; MNSc marie.stenberg@mau.se

Elisabeth Mangrio[1] RN, PhD, Associate Professor elisabeth.mangrio@mau.se

Mariette Bengtsson[1] RN, PhD, Associate Professor mariette.bengtsson@mau.se

Elisabeth Carlson[1] RN, PhD, Professor elisabeth.carlson@mau.se

[1]Department of Care Science, Faculty of Health and Society, Malmö University, SE 205 06 Sweden

Corresponding author: Marie Stenberg, Faculty of Health and Society, Jan Waldenströmsg 25, Malmö University, SE 214 28 Malmö, Sweden

Tel: +46 40 665 79 44

E-Mail: marie.stenberg@mau.se

Word count: 3533

For peer review only

ABSTRACT

Background: Formative peer assessment focus learning and development of the student learning process. This implies students taking responsibility for assessing the work of their peers by giving and receiving feedback to each other. Previous reviews seems to lack compiled research considering formative peer assessment in its entirety. The aim of the current systematic scoping review was to compile research about formative peer assessment presented in higher healthcare education, focusing the rationale, the interventions, the experiences of students and teachers and the outcomes of formative assessment interventions.

Method: The systematic search in four databases resulted in 1452 studies were 37 met the inclusion criteria and were critically appraised. The critical appraisal resulted in 18 included studies with high and moderate quality. The pertinent data was analyzed using thematic analysis.

Result: The results present clinical skill-training courses to be a frequent setting for formative peer assessment activities, focusing intra-professional peers. The rationale for using formative peer assessment relates to giving and receiving constructive feedback as a means to promote learning. The experience and outcome of formative peer assessment interventions from the perspective of students and teachers is presented within three themes; 1/the organization and structure of the formative peer assessment activities, 2/ personal attributes and consequences for one self and relationships and 3/ the experience and outcome of feedback and learning.

Conclusion: Healthcare education must consider preparing and introducing students to collaborative learning and thus develop of well-designed learning activities aligned with the learning outcomes. For formative peer assessment to be effective it needs to be implemented in a collaborative learning environment. Since peer collaboration seems to affect students' and teachers' experiences of formative peer assessment empirical investigations exploring collaboration between students is of utmost importance.

Keywords: Feedback, formative assessment, healthcare education, peer assessment, students, teachers

For peer review only

For peer review only - http://bmjopen.bmj.com/site/about/guidelines.xhtml

STRENGTHS AND LIMITATIONS OF THE STUDY

- The current scoping review is previously presented in a published study protocol.
- Four databases were systematically searched to identify research on formative peer assessment.
- Critical appraisal tools were used to assess the quality of studies with quantitative, qualitative, and mixed methods designs.
- Articles appraised with high or moderate quality were included.
- Since only English studies were included, studies may have been missed that would otherwise have met the inclusion criteria.

BACKGROUND

Peer assessment is an educational approach where feedback, communication, reflection, and collaboration between peers are key characteristics. In a peer assessment activity, students take responsibility for assessing the work of their peers by giving (and receiving) feedback on a specific subject.[1] It allows students to consider the learning outcomes for peers of similar status and to reflect upon their own learning mirrored in a peer.[2] Peer assessment has shown to support students' development of judgement skills, critiquing abilities, and self-awareness as well as their understanding of the assessment criteria used in a course.[1] In higher education, peer assessment has been a way to move from an individualistic and teacher-led approach to a more collaborative, student-centred approach to assessment[1] aligned with social constructivism principles.[3] In this social context of interaction and collaboration, students can expand their knowledge, identify their strengths and weaknesses, and develop personal and professional skills[4] by evaluating the professional competence of a peer.[5] Peer assessment can be used in academic and professional settings as a strategy to enhance students' engagement in their own learning.[6 7 8] The collaborative aspect of peer assessment relates to professional teamwork, as well as to broader goals for lifelong learning. As argued by Boud et al.,[1] peer assessment addresses course-specific goals not readily developed otherwise. For healthcare professions, it enhances ability to work in a team in a supportive and respectful atmosphere,[9] which is highly relevant for patient outcome and the reduction of errors for patient safety.[10] However, recent research has shown that peer collaboration is challenging[11] and that healthcare professionals are not prepared to deliver and receive feedback effectively.[12] This emphasizes the importance for healthcare educators to support students with activities fostering these competences. Feedback is highly associated with enhancing student learning[13]

For peer review only - http://bmjopen.bmj.com/site/about/guidelines.xhtml

and modifying learning during the learning process[14] so students can close the gap between their present state of learning and their desired goal(s). Peer feedback can be written or oral, conducted as peer observations in small or large groups.[8] Further, it is driven by set assessment criteria,[1] which can be either summative or formative, formal or informal. Summative assessment evaluates students' success or failure after the learning process,[15] whereas formative assessment aims for improvement during the learning process.[16,4] According to Black and Wiliam,[15] formative peer assessment activities involve feedback to modify the teaching and learning of the students. The intention of feedback is to help students help each other when planning their learning.[4] [17] An informal formative peer assessment activity involves a continuous process throughout a course or education, whereas a formal one is designated to a single point in a course's momentum. Earlier research on peer assessment in healthcare education has provided an overview of specific areas within the peer assessment process. For example, Speyer, Pilz, and Van Der Kruis presented psychometric characteristics of peer assessment instruments and questionnaires in medical education,[18] concluding that quite a few instruments exist; however, these intruments mainly focus on professional behavior, and they lack sufficient psychometric data. Tornwall[19] focused on how nursing students are prepared by academics to participate in peer assessment activities and highlighted the importance of creating a supporting learning environment. Lerchenfeldt, Mi and Eng[20] concluded that peer assessment supports medical students in developing professional behavior and that peer feedback is a way to assess professionalism. Khan, Payne, and Chahine[21] reviewed the role of peer assessment in objective structured clinical examination (OSCE), showing that peer assessment promotes learning but that students need training in how to provide feedback. In short, the existing literature contributes valuable knowledge about formative peer assessment in healthcare education targeting specific areas. However, there seems to be lack of compiled research considering formative peer assessment in its entirety, including the context, rationale, experience, and outcome of the formative peer assessment process. Therefore, this scoping review attempts to present an overview of formative peer assessment in healthcare education rather than specific areas within that process.

METHOD

This systematic scoping review was conducted using the York methodology by Arksey and O'Malley [22] and the recommendations presented by Levac, Colquhuan, and O'Brien.[23] We constructed a scoping protocol, utilizing a PRISMA-P protocol, to present the planned methodology for the scoping review.[24]

For peer review only - http://bmjopen.bmj.com/site/about/guidelines.xhtml

Aim and research questions

We aimed to compile research about formative peer assessment presented in higher healthcare education. The research questions were as follows: What are the rationales for using formative peer assessment in healthcare education? How are formative peer assessment interventions delivered in healthcare education and in what context? What experiences of formative peer assessment do students and teachers in healthcare education have? What are the outcomes of formative peer assessment interventions? We used the "Population Concept and Context" (PCC) elements recommended for scoping reviews to establish effective search criteria (Table 1).[25]

INSERT TABLE 1 HERE

Relevant studies identified

The literature search was conducted in the databases PubMed, Cumulative Index to Nursing and Allied Health Literature (CINAHL), Education Research Complete (ERIC), and Education Research Centre (ERC). Search tools such as Medical Subject Headings, Headings, Thesaurus and Boolean operators (AND/OR) helped expand and narrow the search. Initially, the search terms were broad (e.g., peer assessment or higher education) in order to capture the range of published literature. However, the extensiveness of material made it necessary to narrow the search terms and organize them in three major blocks. The following inclusion criteria were applied in the search: (a) articles addressing formative peer assessment in higher education; (b) students and teachers in medical-nursing, midwifery, dentistry, physical or occupational therapy, and radiology; and (c) peer reviewed articles, grey literature (books, discussion papers, posters et cetera). Studies of summative peer assessment, instrument development, and systematic reviews were excluded. We incorporated several similar terms related to peer assessment in the search to ensure that no studies were missed (Appendix 1). Furthermore, we consulted a well-versed librarian with experience of systematic search[26] to assist us systematically identify relevant search terms in each database, control the relevance of the constructed search blocks, and manage the data in a reference management system.

Study selection

The process of the study selection and the reasons for exclusion are presented in a flow diagram[27] (Figure 1). First, the first author (MS) screened all 1,452 titles. Second, MS read all the abstracts, gave those responding to the research questions a unique code, and organized

For peer review only - http://bmjopen.bmj.com/site/about/guidelines.xhtml

them in a reference management system. An additional hand search of reference lists was conducted. To cover a subject in full, a scoping review should include search in grey literature.[22 23] Therefore, the grey literature was scoped to find unpublished results by searching Google Scholar, LibSearch, and Science Direct. The grey literature mostly contained research posters, conference abstracts, discussion papers, and books, but a hand search revealed original research articles that were added for further screening and appraisal. Finally, the first author (MS) arrived at 81 studies, read them in full-text, and discussed them with the other three authors (EC, MB, EM).

INSERT FIGURE 1 HERE

Charting the data

We constructed a charting form to facilitate the screening of the full-text studies (Appendix 2). Out of the 81 studies, 37 met the inclusion criteria and were appraised for quality using Critical Appraisal Skills Programme (CASP).[28] To investigate the interpretation of the quality instrument, three members of the research team (MS, EM, EC) conducted an initial test assessment of two randomly selected studies and graded them with high, moderate, or low quality. Additional screening tools were used for studies with a mixed methods design[29] and cross-sectional studies[30] not available in CASP. When a discrepancy arose, a fourth researcher (MB) assessed the articles independently without prior knowledge of what the others have concluded. This was followed by a discussion among all four researchers to secure internal agreement on how to further interpret the checklist items and quality assessment. Consequently, to ensure high quality, the studies had to have a "yes" answer for a majority of the questions. If "no" dominated, the study was excluded. Since earlier reports[31] have raised and discussed the importance of ethical issues in systematic reviews, all screening protocols in this review included ethical considerations, as an individual criterion. The first author critically appraised all 37 articles, and 15 articles were divided between the team members (EC, MB, EM) and independently appraised. The screening process was conducted using the Rayyan system for systematic reviews, presented by Ouzzani et al.[32] The critical appraisal resulted in 18 studies with high and moderate quality (Table 2).

INSERT TABLE 2 HERE

Collating, summarizing, and reporting results

The analysis process followed the five phases of thematic analysis described by Braun and Clarke,[33] with support of a practical guide provided by Maquire & Delahunt.[34] The first phase

For peer review only - http://bmjopen.bmj.com/site/about/guidelines.xhtml

includeed familiarizing with the data. Therefore, prior to the coding process, we read all the articles to grasp a first impression of the results presented within the included studies.We then conducted a theoretical thematic analysis, meaning that the results were deductively coded,[33] guided by the research questions. We read the results a second time before starting the initial coding. The codes consisted of short descriptions close to the original text. The codes were then combined into themes and sub-themes. The themes were identified with a semantic approach, meaning that they were explicit: we did not look for anything beyond what was written.[33] Finally, we constructed a thematic map to present an overview of the results and how they related to each other. The results from the studies are presented narratively.

Consultation

Consultation is an optional stage in scoping reviews.[22] However, since it adds methodologic rigor,[23] we presented and discussed the preliminary results and the thematic map with nine academic teachers, experts within the field of healthcare education and pedagogy. The purpose of the consultation was to enhance the validity of the results of the scoping review and to facilitate appropriate dissemination of outputs.[33] The expert group responded to four questions: Do the themes make sense? Is too much data included in one single theme? Are the themes distinct or do they overlap? Are there themes within themes?[34] The consultation resulted in a revision of a few themes and the way they related to each other.

RESULTS

The 18 included studies were published between 2002 and 2017 in the United States (6), the United Kingdom (6), Australia (3), Canada (2), and the United Arab Emirate (1) (Table 3). The studies were conducted in undergraduate medical (12), dental (2), nursing (2), occupational therapy (1), and radiography (1) educations. Six studies were presented in the framework of an existing collaborative educational model.[35-37 38 39 40] Our review revealed that the most frequent setting for formative peer assessment activities is within clinical skill-training courses,[35 39-47] involving intra-professional peers. The common rationale for using formative peer assessment is to support students, usually explained by the inherent learning of the feedback process,[35 39 40 43-45 47-51] and to prepare students for professional behavior and provide them with the skills required in the health care professions.[36 37 38 46 47 48 49 52] Table 3 presents the results of the analysis related to the research questions of context, rationale, and interventions of formative peer assessment.

For peer review only - http://bmjopen.bmj.com/site/about/guidelines.xhtml

INSERT TABLE 3 HERE

The results related to the research questions about the experience of students and teachers and the outcome of formative peer assessment interventions fall within three themes: (1) the organization and structure of peer assessment activities, (2) personal attributes and consequences for oneself and one's peer relationships, and (3) the experience and outcome of feedback and learning.

The organization and structure of formative peer assessment activities

In the reviewed studies, students express viewing the responsibility of faculty as a key component in formative peer assessment, meaning that faculty must clearly state the aim of the peer assessment activity. Students highlight the need to be prepared and trained in how to give and receive constructive feedback.[36 47 50-52] The learning activities need to be well-designed and supported by guidelines on how to use them.[35 36 50 52] Otherwise, it could discourage students from participating in the peer activities.[52] Novice students find it difficult to be objective and to offer constructive criticism in a group.[36 46] This emphasizes the importance of responsibility from faculty, especially when students are to give feedback on professional behavior.[52] Some students prefer direct communication with peers when feedback is negative, whereas others think it is the responsibility of faculty.[52] There is some ambiguity regarding whether feedback should be given anonymously or not,[47 52] whether it should bear consequences from faculty or not,[52] whether it should be informal or formal, and whether the peer should be at the same academic level or at a more experienced higher-level.[50 52] However, some students express favoring small groups;[41 49] further, students in small groups show more activity than those in large groups.[41] Students and teachers agree that peer assessment should be strictly formative rather than summative.[42 46 52] Teachers see themselves as key facilitators and express that students value feedback from teachers rather than from peers (in terms of credibility).[51] Students express similar sentiments even if they appreciated the peer feedback.[40 42 44 46] However, teachers confirm the need for training and preparing students early in the education, as well as the need for their own professional development to guide students effectively.[51]

Personal attributes and the impact and consequences for oneself and one's peer relationships

For peer review only - http://bmjopen.bmj.com/site/about/guidelines.xhtml

For peer review only

Students generally focus on how peer assessment activities may affect their personal relationships in a negative way.[35 37 42 50 52] They express worry over consequences for themselves and their social relationships[37 40 52] as well as anxiety that negative feedback given to a peer may affect the grading from faculty.[52] Moreover, students emphasize the importance of enthusiasm and engagement in listening to peers' opinions during their collaboration.[36 47] They mention positive personal attributes and behaviors such as being organized, polite, and helpful as a support for peer collaboration.[36 47] Further, they mention the importance of both a positive and close relationship between students and faculty[52] and a positive culture in the learning environment.[40] While students highlight the impact and consequences on personal relationships, teachers speak of the importance of respect in formative peer assessment,[36] including respect for each other, the learning activity, and the collaboration and interaction.[36] Further, teachers emphasize the importance of students being self-aware, being well prepared, and taking own responsibility for the peer assessment activity.[36]

The experience and outcome of feedback and learning

According to the students in the reviewed studies, formative peer assessment contributes to developing the skills needed in practice and in their future profession.[35 36 40 41 48 52] They appreciate the opportunity to give and receive feedback from a peer,[35 36 40 42 47 48 50] and they agree that the feedback they received made them change how they worked[42 48] or how they taught their peers.[47 48] They consider activities such as observation of others' performance as beneficial for learning because they make them reflect on their own performance[35 36 40 41 46 49 50] and help them identify knowledge gaps.[35 40 49] Students with prior experience of peer learning are more likely to provide specific guiding feedback than those without such experiences.[39] Moreover, two studies showed significantly improved test results for students who took part in a peer feedback activity compared to those who did not.[43 49] Further, students think they could be honest in their feedback and would learn better if the feedback was more in-depth.[35 46] Students at entry level tend to give more positive feedback than senior students; they also focus on practical and clinical knowledge, whereas students in year five focus on communication, management, and leadership in their feedback comments.[45] A study exploring what students remember of received feedback shows they remember positive growth, negative self-image, and negative attitudes toward classmates. Received feedback sometimes confirmed personal traits the students already knew about.[37] In addition, negative feedback was more likely to

For peer review only - http://bmjopen.bmj.com/site/about/guidelines.xhtml

result in a change in their work habits and interpersonal attributes.[37] Students express some anxiety regarding the usefulness of feedback from low-performing students[40 50] and non-motivated students, which contributes to ineffective interaction and learning.[36 47] Low performing students showlack of initiative, preparation, and respect but also show improvement in their ratings after the peer assessment experience.[47] Furthermore, feedback from peers can be a predictor of a student's unprofessional behavior; hence, it could be used as a tool for early remediation.[38] In an evaluation of faculty examiners' experience of students' feedback, the faculty express they view student feedback to be given in a professional and appropriate way and faculty examiners would have given similar feedback.[42] In an OSCE-examination where a checklist was used, the results show statistical significance in assessment between faculty examiners and student examiners.[42]

DISCUSSION

We conclude that formative peer assessment is a process with two consecutive phases. The first phase concerns the understanding of the rationale and fundament of the peer assessment process for students and faculty members. The results indicate that the rationale is to support student learning and prepare them for healthcare professions. Nevertheless, students express the importance of clarifying the aim of the peer assessment activity and the responsibility of the faculty. We recommend faculty to clearly define the activity and explain how it supports student learning and professionalism, especially when students are to provide feedback to each other on sensitive matters, such as un-professional behavior. Otherwise, there is a risk that students might perceive peer assessment as an activity meant to ease the burden on the teachers.[53 54] A collaborative activity requires trust, and the real intention must be made transparent. [4 55-57] Moreover, to enable student development in-line with the learning outcomes, the learning activity needs to be well designed and understood by students so they can advantageously relate to the purpose.[58 59 60] However, Casey et al.[61] recommended further investigations of how to prepare students for the peer assessment activities.

The second phase concerns the organization and structure of the formative peer assessment activity, for example, how to give and receive feedback. The current scoping review reveals the complexity of peer collaboration in formative peer assessment: It affects students' emotions concerning both themselves and their relationship with their peers. This coincides with earlier research emphasizing the social factors of peer assessment and the importance for teachers to consider them.[4] Nevertheless, surprisingly, few studies highlight the collaborative

For peer review only - http://bmjopen.bmj.com/site/about/guidelines.xhtml

part of peer assessment.[4 11] One reason might be that formative peer assessment is often presented as a "stand alone" activity and not involved in a collaborative learning environment.[8 62] We agree with earlier research[63 64] arguing that peer assessment needs to be affiliated with practices of collaborative learning. Similar implications are presented by Tornwall,[19] who concluded the importance of integrating peer collaboration as a natural approach throughout education to support student development.

CONCLUSIONS AND IMPLICATIONS FOR FURTHER RESEARCH

Some have argued that the research on peer assessment is deficient in referring to exactly what peer assessment aims to achieve.[65] We conclude that within healthcare education the aim of formative peer assessment is to prepare students for the collaborative aspects crucial within the healthcare professions. However, healthcare education must consider preparing and introducing students to collaborative learning; therefore, it must develop well-designed learning activities aligned with the learning outcomes. Based on this systematic scoping review, formative peer assessment needs to be implemented in a collaborative learning environment throughout the education to be effective. However, since peer collaboration seems to affect students' and teachers' experiences of formative peer assessment, empirical investigations exploring the collaboration between students are of utmost importance.

LIMITATIONS

Previous methodological concerns and discussions have been related to the systematic approach of handling grey literature.[66 67] We argue that the grey literature contributes to a wider understanding of the research area. When we were conducting a critical appraisal of included studies, the grey literature was excluded due to lack of methodological rigor. Therefore, we recommend considering this time-consuming phase of the methodology in systematic scoping reviews. Further, the current scoping review has not fully elucidated the perspective of teachers and faculty. Few of the included studies highlight the teachers' perspective, so further research is required.

Authors' contribution: MS led the design, search strategy, and conceptualization of this work and drafted the manuscript. EM, MB, and EC were involved in the conceptualization of the review design, inclusion and exclusion criteria, and critical appraisal and provided feedback on the methodology and the manuscript. All authors give their approval to the publishing of this scoping review manuscript.

For peer review only - http://bmjopen.bmj.com/site/about/guidelines.xhtml

Funding statement: This research received no specific grant from any funding agency in the public, commercial, or not-for-profit sectors.

Competing interests: None declared.

Patient and public involvement: As this is a scoping review no primary research has been conducted and thus not involve patients or the public.

Acknowledgement: Special thanks to the members in the expert group for their valuable contribution in the consultation.

REFERENCES

1. Boud D, Cohen R, Sampson J. Peer learning and assessment. *Assessment & evaluation in higher education* 1999;24(4):413-26.
2. Topping KJ, Ehly SW. Peer assisted learning: A framework for consultation. *Journal of Educational and Psychological Consultation* 2001;12(2):113-32.
3. Vygotskiĭ LS. Thought and language: MIT press 2012.
4. Topping KJ. Peer Assessment. 2009:20-27. doi: 10.1080/00405840802577569
5. Dannefer EF, Henson LC, Bierer SB, et al. Peer assessment of professional competence. *Medical Education* 2005;39(7):713-22. doi: 10.1111/j.1365-2929.2005.02193.x
6. Casey D, Burke E, Houghton C, et al. Use of peer assessment as a student engagement strategy in nurse education. *Nursing & health sciences* 2011;13(4):514-20.
7. Orsmond* P, Merry S, Callaghan A. Implementation of a formative assessment model incorporating peer and self-assessment. *Innovations in Education and Teaching International* 2004;41(3):273-90.
8. Ashenafi MM. Peer-assessment in higher education – twenty-first century practices, challenges and the way forward. *Assessment and Evaluation in Higher Education* 2017;42(2):226-51. doi: 10.1080/02602938.2015.1100711
9. Sims S, Hewitt G, Harris R. Evidence of collaboration, pooling of resources, learning and role blurring in interprofessional healthcare teams: a realist synthesis. *Journal of Interprofessional care* 2015;29(1):20-25.
10. Morley L, Cashell A. Collaboration in health care. *Journal of medical imaging and radiation sciences* 2017;48(2):207-16.
11. Stenberg M, Carlson E. Swedish student nurses' perception of peer learning as an educational model during clinical practice in a hospital setting—an evaluation study. *BMC nursing* 2015;14(1):48.
12. Tornwall J. Peer assessment practices in nurse education: An integrative review. *Nurse education today* 2018;71:266-75.
13. Nicol DJ, Macfarlane-Dick D. Formative assessment and self-regulated learning: A model and seven principles of good feedback practice. *Studies in higher education* 2006;31(2):199-218.
14. Hudson JN, Bristow DR. Formative assessment can be fun as well as educational. *Advances in physiology education* 2006;30(1):33-37. doi: 10.1152/advan.00040.2005
15. Black P, Wiliam D. Assessment and classroom learning. *Assessment in Education: principles, policy & practice* 1998;5(1):7-74.
16. Sadler DR. Beyond feedback: Developing student capability in complex appraisal. *Assessment & Evaluation in Higher Education* 2010;35(5):535-50.
17. Topping K. Peer assessment between students in colleges and universities. *Review of educational Research* 1998;68(3):249-76.

For peer review only - http://bmjopen.bmj.com/site/about/guidelines.xhtml

18. Speyer R, Pilz W, Van Der Kruis J, et al. Reliability and validity of student peer assessment in medical education: a systematic review. *Med Teach* 2011;33(11):e572-85. doi: 10.3109/0142159X.2011.610835 [published Online First: 2011/10/26]

19. Tornwall J. Peer assessment practices in nurse education: An integrative review, 2018:266-75.

20. Lerchenfeldt S, Mi M, Eng M. The utilization of peer feedback during collaborative learning in undergraduate medical education: a systematic review. *BMC medical education* 2019;19(1):321.

21. Khan R, Payne MWC, Chahine S. Peer assessment in the objective structured clinical examination: A scoping review. *Medical Teacher* 2017;39(7):745-56. doi: 10.1080/0142159X.2017.1309375

22. Arksey H, O'Malley L. Scoping studies: towards a methodological framework. *International Journal of Social Research Methodology* 2005;8(1):19-32. doi: 10.1080/1364557032000119616

23. Levac D, Colquhoun H, O'Brien KK. Scoping studies: advancing the methodology. *Implementation Science* 2010;5(1):69. doi: 10.1186/1748-5908-5-69

24. Stenberg M, Mangrio E, Bengtsson M, et al. Formative peer assessment in healthcare education programmes: protocol for a scoping review. *BMJ open* 2018;8(11):e025055-e55. doi: 10.1136/bmjopen-2018-025055

25. Peters M, Godfrey C, McInerney P, et al. Methodology for JBI scoping reviews. The Joanna Briggs Institute Reviewers manual 2015: The Joanna Briggs Institute 2015:3-24.

26. Morris M, Boruff JT, Gore GC. Scoping reviews: establishing the role of the librarian. *Journal of the Medical Library Association: JMLA* 2016;104(4):346.

27. Moher D, Liberati A, Tetzlaff J, et al. Preferred reporting items for systematic reviews and meta-analyses: the PRISMA statement. *Annals of internal medicine* 2009;151(4):264-69.

28. Programme CAS. Critical Appraisal Skills Programme. CASP checklist 2018 [Available from: https://casp-uk.net/casp-tools-checklists/ accessed 5 august 2018.

29. Hong Q, Pluye P, Fàbregues S, et al. Mixed Methods Appraisal Tool (MMAT) version 2018: User guide. *Department of Family Medicine, McGuill Univertiy* 2018

30. Institute JB. Critical Appraisal Tools [Available from: http://joannabriggs.org/research/critical-appraisal-tools.html accessed 5 august 2018.

31. Weingarten MA, Paul M, Leibovici L. Assessing ethics of trials in systematic reviews. *Bmj* 2004;328(7446):1013-14.

32. Ouzzani M, Hammady H, Fedorowicz Z, et al. Rayyan—a web and mobile app for systematic reviews. *Systematic reviews* 2016;5(1):210.

33. Braun V, Clarke V. Using thematic analysis in psychology. *Qualitative research in psychology* 2006;3(2):77-101.

34. Maguire M, Delahunt B. Doing a thematic analysis: A practical, step-by-step guide for learning and teaching scholars. *AISHE-J: The All Ireland Journal of Teaching and Learning in Higher Education* 2017;9(3)

35. Cushing A, Abbott S, Lothian D, et al. Peer feedback as an aid to learning - What do we want? Feedback. When do we want it? Now! *Medical Teacher* 2011;33(2) doi: 10.3109/0142159X.2011.542522

36. Iqbal M, Velan GM, O'Sullivan AJ, et al. Differential impact of student behaviours on group interaction and collaborative learning: medical students' and tutors' perspectives. *BMC medical education* 2016;16(1):217-17. doi: 10.1186/s12909-016-0730-1

37. Nofziger AC, Naumburg EH, Davis BJ, et al. Impact of peer assessment on the professional development of medical students: a qualitative study. *Academic medicine : journal of the Association of American Medical Colleges* 2010;85(1):140-47. doi: 10.1097/ACM.0b013e3181c47a5b

38. Emke AR, Cheng S, Chen L, et al. A Novel Approach to Assessing Professionalism in Preclinical Medical Students Using Multisource Feedback Through Paired Self- and Peer Evaluations. *Teaching and learning in medicine* 2017;29(4):402-10. doi: 10.1080/10401334.2017.1306446

For peer review only

For peer review only - http://bmjopen.bmj.com/site/about/guidelines.xhtml

39. Chou CL, Masters DE, Chang A, et al. Effects of longitudinal small-group learning on delivery and receipt of communication skills feedback. *Medical Education* 2013;47(11):1073-79. doi: 10.1111/medu.12246
40. Tai JH-M, Canny BJ, Haines TP, et al. The role of peer-assisted learning in building evaluative judgement: opportunities in clinical medical education. *Advances in health sciences education : theory and practice* 2016;21(3):659-76. doi: 10.1007/s10459-015-9659-0
41. Cho Y, Je S, Yoon YS, et al. The effect of peer-group size on the delivery of feedback in basic life support refresher training: A cluster randomized controlled trial. *BMC Medical Education* 2016;16(1):1-8. doi: 10.1186/s12909-016-0682-5
42. Moineau G, Power B, Pion A-MJ, et al. Comparison of student examiner to faculty examiner scoring and feedback in an OSCE. *Medical education* 2011;45(2):183-91. doi: 10.1111/j.1365-2923.2010.03800.x
43. Satterthwaite JD, Grey NJA. Peer-group assessment of pre-clinical operative skills in restorative dentistry and comparison with experienced assessors. *European Journal of Dental Education* 2008;12(2):99-102. doi: 10.1111/j.1600-0579.2008.00509.x
44. Vaughn CJ, Kim E, O'Sullivan P, et al. Peer video review and feedback improve performance in basic surgical skills. *American Journal of Surgery* 2016;211(2):355-60. doi: 10.1016/j.amjsurg.2015.08.034
45. Tricio J, Woolford M, Escudier M. Analysis of dental students' written peer feedback from a prospective peer assessment protocol. *European Journal of Dental Education* 2016;20(4):241-47. doi: 10.1111/eje.12187
46. Rees C, Sheard C, McPherson A. Communication skills assessment: the perceptions of medical students at the University of Nottingham. *Medical Education* 2002;36(9):868-78.
47. Spandorfer J, Puklus T, Rose V, et al. Peer assessment among first year medical students in anatomy. *Anatomical sciences education* 2014;7(2):144-52. doi: 10.1002/ase.1394
48. Martin D, Friesen E, De Pau A. Three heads are better than one: a mixed methods study examining collaborative versus traditional test-taking with nursing students. *Nurse education today* 2014;34(6):971-77. doi: 10.1016/j.nedt.2014.01.004
49. Mui Lim S, Rodger S. The use of interactive formative assessments with first-year occupational therapy students. *International Journal of Therapy and Rehabilitation* 2010;17(11):576-86.
50. Elshami W, Abdalla ME. Diagnostic radiography students' perceptions of formative peer assessment within a radiographic technique module. *Radiography (London, England : 1995)* 2017;23(1):9-13. doi: 10.1016/j.radi.2016.06.001
51. Koh LC. Academic staff perspectives of formative assessment in nurse education. *Nurse education in practice* 2010;10(4):205-09. doi: 10.1016/j.nepr.2009.08.007
52. Arnold L, Shue CK, Kritt B, et al. Medical students' views on peer assessment of professionalism. *Journal of General Internal Medicine* 2005;20(9):819-24. doi: 10.1111/j.1525-1497.2005.0162.x
53. Cassidy S. Assessing 'inexperienced'students' ability to self-assess: Exploring links with learning style and academic personal control. *Assessment & Evaluation in Higher Education* 2007;32(3):313-30.
54. Thomas G, Martin D, Pleasants K. Using self-and peer-assessment to enhance students' future-learning in higher education. *Journal of University Teaching and Learning Practice* 2011;8(1):5.
55. Vickerman P. Student perspectives on formative peer assessment : an attempt to deepen learning ? 2009;34(2):221-30. doi: 10.1080/02602930801955986
56. Sluijsmans D, Prins F. A conceptual framework for integrating peer assessment in teacher education. *Studies in Educational Evaluation* 2006;32(1):6-22.
57. Boud D, Falchikov N. Developing assessment for informing judgement. *Rethinking assessment in higher education: Learning for the longer term* 2007:181-97.
58. Cassidy S. Developing employability skills : peer assessment in higher education. 2006 doi: 10.1108/00400910610705890

For peer review only - http://bmjopen.bmj.com/site/about/guidelines.xhtml

59. Solheim E, Plathe HS, Eide H. Nursing students' evaluation of a new feedback and reflection tool for use in high-fidelity simulation – Formative assessment of clinical skills. A descriptive quantitative research design. *Nurse Education in Practice* 2017;27:114-20. doi: 10.1016/j.nepr.2017.08.021
60. Topping KJ. Peer assessment. *Theory into practice* 2009;48(1):20-27.
61. Casey D, Burke E, Houghton C, et al. Use of peer assessment as a student engagement strategy in nurse education. *Nursing and Health Sciences* 2011;13(4):514-20. doi: 10.1111/j.1442-2018.2011.00637.x
62. Papinczak T, Young L, Groves M, et al. An analysis of peer, self, and tutor assessment in problem-based learning tutorials. *Med Teach* 2007;29(5):e122-32. doi: 10.1080/01421590701294323 [published Online First: 2007/09/22]
63. Kollar I, Fischer F. Peer assessment as collaborative learning: A cognitive perspective. *Learning and Instruction* 2010;20(4):344-48.
64. Finn GM, Garner J. Twelve tips for implementing a successful peer assessment. *Medical teacher* 2011;33(6):443-46.
65. Jackel B, Pearce J, Radloff A, et al. Assessment and feedback in higher education.
66. Gentles SJ, Lokker C, McKibbon KA. Health information technology to facilitate communication involving health care providers, caregivers, and pediatric patients: a scoping review. *Journal of medical Internet research* 2010;12(2):e22.
67. Pham MT, Rajić A, Greig JD, et al. A scoping review of scoping reviews: advancing the approach and enhancing the consistency. *Research synthesis methods* 2014;5(4):371-85.

For peer review only

For peer review only - http://bmjopen.bmj.com/site/about/guidelines.xhtml

Table 1. The PCC mnemonic as recommended by the Joanna Briggs Institute

Population	Concept	Context
Students assessing students	Intervention, rationale, outcome, context, and students' and teachers' experience of formative peer assessment	Healthcare education programs in higher education

For peer review only

For peer review only - http://bmjopen.bmj.com/site/about/guidelines.xhtml

Table 2. Overview of included studies.

**Author, year, country, and journal**	**Aim**	**Design**	**Participants**	**Main findings**	**Quality***
Arnold et al., 2005,[52] USA<br><br>*Journal of General Internal Medicine*	Identify factors that encourage or discourage student participation in peer assessment	Qualitative<br>Grounded theory<br><br>Focus groups (16) at two medical schools	n=61, medical students in year 1, 3, and 4	The characteristics of the peer assessment system and the environment can encourage or discourage participation. Themes: (1) Students' struggle with peer assessment, (2) Characteristics of a peer assessment system, and (3) Environmental factors	moderate
Cho et al., 2016,[41] England<br><br>*BMC Medical Education*	Investigate the effect of peer-group size on competency-based skills	Quantitative<br>Cluster RCT	n=115, medical students in year 6	Smaller groups (4.1) show more active and preferred than large groups (8,1). Group size did not impact scores.	high
Chou et al., 2013,[39] USA<br><br>*Medical Education*	Examine the role of prior peer-learning relationships between students in their delivery and receipt of feedback on clinical communication skills	Mixed method<br>Case-control<br>Descriptive statistics<br>Survey, video observations	n= 72 medical students in year 3 with prior peer learning relationships<br><br>n=36 students in control group with no prior peer relationships.	Students with prior peer learning relationships more likely to provide specific corrective feedback than those without prior relationships. No significant difference between groups regarding how feedback was received.	moderate
Cushing et al., 2011,[35] United Kingdom<br><br>*Medical Teacher*	Investigate the benefits of formative peer feedback in communication skills and develop a training programme in peer feedback	Mixed method<br>Questionnaire (20 items) at two occasions with 6 months in between.<br>Focus groups (5 medical- and 2 nurse students)	n=45 medical students in year 1<br><br>n=48 nursing students in year 1	Students valued the learning opportunity of both being examiner and observer. They preferred more in-depth feedback and feedback from tutors. They expressed anxiety about giving negative feedback to a peer and had mixed views on giving feedback (relaxed or pressured) and its use in clinical placements.	high
Elshami & Abdalla, 2017,[50]<br>United Arab Emirates<br><br>*Radiography*	Assess perception of formative peer assessment	Qualitative<br>Action research<br>Focus groups (3)<br>Content analysis	n=19 (24**) diagnostic radiography students in year 3	Formative peer assessment gives valuable feedback from same level or more experienced peers. Need for training and detailed rubrics.	moderate
Emke et al., 2017,[38] USA	Demonstrate that perceptual errors related to professionalism behaviors can be detected early through	Quantitative	n=246 medical students in year 2	Multiple peer assessments and feedback a tool predictor of unprofessional behavior.	moderate

For peer review only - http://bmjopen.bmj.com/site/about/guidelines.xhtml

Teaching and Learning in Medicine	repeated multisource feedback				
Iqbal et al., 2016,[36] Australia<br><br>*BMC Medical Education*	Explore students' and tutors' perception of key collaborative behaviors that impact collaborative learning and interaction	Qualitative<br>Focus groups (5) with students<br>Interviews (8) with teachers<br>Thematic analysis	n= 22 medical students in year one and two<br><br>n= 8 teachers	Being respectful, giving constructive feedback, and being engaged and prepared had positive impact on both learning and group interaction. Passiveness, unreliability, irresponsibility, and condescending attitudes had a negative impact on learning and interaction. Similar results from teachers.	high
Koh, 2010,[51] United Kingdom<br><br>*Nurse Education in Practice*	Explore how academic staff experience, understand, and interpret the process of formative assessment and feedback of theoretical assessment	Qualitative<br>Phenomenology<br>Semi-structured interviews (22)<br>Thematic analysis	n=20 academic staff in nurse education	Teachers see themselves as key facilitators and think students prefer teacher feedback. Students are assumed to have the skill to peer assess and give feedback but are unprepared and need support and introduction early in education. Teachers need professional development themselves.	moderate
Mui Lim & Rodger, 2010.[49] Australia<br><br>*International Journal of Therapy and Rehabilitation*	Improve students learning through interactive formative assessment and student generated questions	Mixed methods<br>Cohort study<br>Evaluation questionnaire	n= 115 occupational therapy students in year 1 in 2009 compared with n= 98 students in 2008	Significant improvement in exams result from being part of interactive formative assessment, which is beneficial for learning and identifying knowledge gaps.	moderate
Martin, Friesen, & De Pau, 2014,[48] Canada<br><br>*Nurse Education Today*	Examine collaborative testing versus traditional test taking with undergraduate nursing students in a nine-station OSCE	Mixed method<br>Cross over design<br>Survey<br>Focus groups	n=70 nursing students	Significantly higher scores in collaborative testing than in traditional testing.<br>Themes: (1) studying more/studying differently, (2)/ cognitive collectivism (3), "it stuck in my head better" (4), confidence, and (5) practicing how to share knowledge and negotiate.	moderate
Moineau et al., 2011,[42] Canada<br><br>*Medical Education*	Compare scores and experiences of formative assessment from faculty and senior students during OSCE-examinations	Quantitative<br>Cross sectional<br>Pre- and post-questionnaire	n=66 medical students in year 2<br>n=27 year 4 student examiners<br>n=27 teaching doctors	Students (year 4) assessing students (year 2) with checklists in OSCE-examinations equally assessed compared to faculty members. A positive learning experience expressed from both students and faculty.	moderate
Nofziger et al., 2010,[37]	Investigate the impact of peer assessment on future	Qualitative	n=70 medical students in year 2	67% found peer assessment helpful, reassuring, or confirming something they	moderate

For peer review only - http://bmjopen.bmj.com/site/about/guidelines.xhtml

USA<br><br>*Academic Medicine*	professional development and students' experiences	Questionnaire and narrative comments<br>Frequency count	n=48 in year 4	knew; 65% reported important transformations in awareness, attitudes, or behaviors because of peer assessment. Change was more likely when feedback was specific and described an area for improvement.	
Rees, Sheard, & McPherson, 2002,[46]<br>United Kingdom<br><br>*Medical Education*	Explore students' perceptions of communication skill assessment	Qualitative<br>Focus groups	n=7 medical students in year 1<br>n= 7 in year 2<br>n=10 in year 3<br>n= 5 in year 4<br>n=3 in year 5	Year 4 and 5 more positive than younger students. Opportunities to compare communication skills with peers from same level. Learning experience being the assessor. No constructive criticism from peers. Difficult to be objective and to give feedback.	high
Satterthwaite & Grey, 2008,[43]<br>United Kingdom<br><br>*European Journal of Dental Education*	Investigate if any differences existed between marks given by a peer group and those given by experienced assessors	Quantitative<br>Cross sectional	n=65 dental students	No significant difference in grades between experienced examiners and peer group.	moderate
Spandorfer et al., 2014,[47]<br>USA<br><br>*Anatomical Science Education*	Determine whether peer assessment improves students work habits and interpersonal attributes and whether it is accepted by students, focusing on low performing students	Multi-methods<br>Paired sample t-test<br>Pearson correlation coefficients<br>Survey- content analysis	n=267 medical students in year 1; follow-up in year 2	Significant improvement after on-line peer feedback between test 1 and 2.<br>Themes: (1) Initiative, (2) Communication, (3) Respect, (4) Preparation, and (5) Focus. Students prefer anonymous feedback from peers.	moderate
Tai et al., 2016,[40]<br>Australia<br><br>*Advances in Health Science Education*	Investigate students' experience of peer-assisted learning.	Mixed methods<br>Ethnographic<br>Survey, observations, and interviews<br>Thematic analysis	n=10 medical students in year 1 (observed)<br><br>n=191 students in year 3 (survey)	Observing and giving feedback to peers contributed to learning, but students value feedback from teachers for validation. Students want to preserve social relationships with peers; therefore, feedback is not so constructive. Peers provide a supportive learning environment.	high
Tricio, Woolford, & Escudier, 2016,[45]<br>United Kingdom<br><br>*European Journal of Dental Education*	Analyze written feedback provided as a part of a formative and structured peer assessment protocol.	Multi-methods<br>Descriptive statistic<br>Thematic analysis	n=40 dental students in year 2 in pre-clinical skills laboratory<br><br>n=68 dental students in year 5 in clinic	Year 2 focuses on practical and clinical knowledge; in contrast, year 5 focuses comments on communication, management, and leadership. Year 2 gives more positive comments on peer performance than year 5.	moderate

For peer review only - http://bmjopen.bmj.com/site/about/guidelines.xhtml

Vaughn et al., 2016,[44] USA<br><br>*The American Journal of Surgery*	Evaluate the use, quality, and quantity of peer video feedback and compare peers and faculty feedback.	Quantitative<br>Cross sectional<br>Paired t-test, Mann-Whitney statistic<br>Survey	n=24 medical students***	Significant change in performance across 3 periods in both groups. Peer feedback group performed better at final assessment than faculty feedback group (not significant). Peers gave higher scores than faculty. No significant differences when using a checklist.	moderate

*High equals majority of items in the critical appraisal tools.

**24 students included in the intervention, and 19 attended the focus group session.

***12 students received faculty feedback, and 12 students received peer feedback.

For peer review only - http://bmjopen.bmj.com/site/about/guidelines.xhtml

Table 3. Overview and summery of the context, rationale, and interventions of formative peer assessment presented in the included studies.

Contexts	Rationales	Interventions
Intra-professional students (17)*<br>Combination of medical and nursing students (1)<br><br>Conducted in the following:<br>Clinical skill labs (11)<br>Theoretical courses (7)<br>Combination of theoretical and clinical placement course (1)<br><br>Within an educational model as problem-based learning, peer learning, or peer assisted learning (7)	Giving and receiving feeback supports student learning:<br>Promotes learning (8)<br>Enhances critical thinking (1)<br>Promotes understanding of the assessment process (1)<br>Develops critical- and interpersonal skills (1)<br>Helps identify knowledge gaps (1)<br>Supports low-performance students (1)<br><br>It prepares students for knowledge-related professionalism in the healthcare profession by helping them identify the following:<br>Professional- and un-professional behavior (6)<br>Clinical competence (2)<br>Technical skills (2)<br>Communication skills (2)<br>Collaborative behavior (2)<br>Evaluative judgement (1)<br><br>It enhances teachers' teaching (1)<br><br>It provides cost benefits:<br>Students as assessors instead of teachers (2)<br>Students as creators of the learning activities instead of teachers (1)	Introduction (in workshops):<br>Preparations in giving and receiving feedback (3)<br>Introduction of guidelines or checklists to guide the peer assessor (3)<br>Introduction of the learning activity (2)<br>Preparation in communication (1)<br><br>Learning activities focusing feedback on professionalism:<br>Clinical skills (3)<br>Collaborative behavior (2)<br>Clinical reasoning (2)<br>Theoretical knowledge (2)<br>Communication skills (2)<br>Management skills (1)<br><br>Feedback types:<br>Face-to-face (7)<br>Anonymous (5)<br>Written (3) or through observations (3)<br>Interactive on-line assessment (3)<br>Grading of the given feedback (1)<br><br>Random peers (8)<br>Ability to choose peer (1)<br>In small groups < 6 (6)<br>In large groups > 6 (3)

* = appears in how many of the included 18 studies

For peer review only

For peer review only - http://bmjopen.bmj.com/site/about/guidelines.xhtml

For peer review only

For peer review only - http://bmjopen.bmj.com/site/about/guidelines.xhtml

Figure 1: PRISMA Flow Diagram

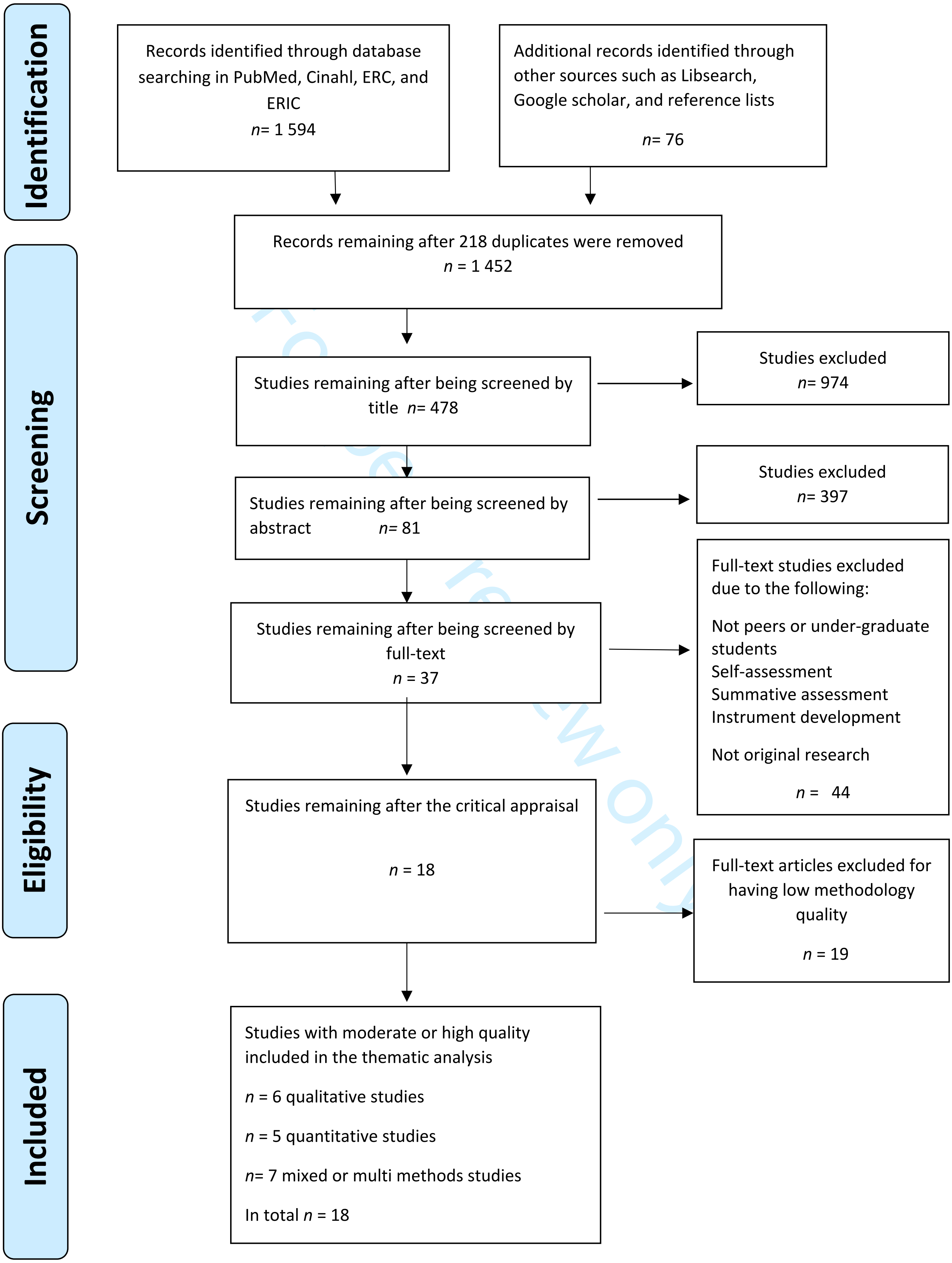

Appendix 1. Presentation of the search strategy and results from the Pub Med database (2019-05-28).

	Search block 1: Healthcare education
#	MESH-terms
1	"Students, Medical"[Mesh] OR
2	"Students, Nursing"[Mesh] OR
3	"Students, Dental"[Mesh] OR
4	"Students, Health Occupations"[Mesh] OR
5	"Education, Medical"[Mesh] OR
6	"Education, Nursing"[Mesh] OR
7	"Education, Dental" [Mesh] OR
8	"Midwifery/education"[Mesh] OR
9	"Allied Health Personnel/education"[Mesh]
10	S1 OR S2 OR S3 OR S4 OR S5 OR S6 OR S7 OR S8 OR S9
#	Free text terms
11	"medical student*" OR
12	"nursing student*" OR
13	"midwifery student* OR
14	"dental student*" OR
15	"physical therapy student*" OR
16	"occupational therapy student*" OR
17	"allied health student*" OR
18	"health occupations student*" OR
19	"health care stud*" OR
20	"Health care education" OR
21	"health science education" OR
22	"Medical education" OR
23	" Nursing education" OR
24	"Dental education" OR
25	"allied health education" OR
26	"Health occupation* education*" OR
27	"midwifery education"
28	S11 OR S12 OR S13 OR S14 ORS15 OR S16 OR S17 OR S18 OR S19 OR S20 OR S21 OR S22 OR S23 OR S24 OR S25 OR S26 OR S27
29	S10 OR S28

	Search block 2: Peer assessment
#	MESH
31	"Educational Measurement"[Mesh] OR
32	"Peer Group"[Mesh] OR
33	"Peer Review"[Mesh]
34	S31 OR S32 OR S33
#	Free text terms
35	"peer assessment" OR
36	"peer evaluation" OR
37	"peer observation" OR
38	"peer feedback" OR
39	"peer review" OR
40	"peer assess*" OR
41	"*peer assess" OR
42	"peer examiner" OR
43	"peer grad*" OR
44	"peer group" OR
45	"Student performance appraisal" OR
46	"educational measurement"
47	S35 OR S36 OR S37 OR S38 OR S39 OR S40 OR S41 OR S42 OR S43 OR S44 OR S45 OR S46
48	S34 OR S47

For peer review only

#	Search block 3: Formative assessment
#	MESH
49	"Formative Feedback"[Mesh]
#	Free text terms
50	"Formative evaluation" OR
51	"Formative feedback" OR
52	“Formative assessment” OR
53	“Formative* assess*” OR
54	“Formativ* evaluation” OR
55	Formativ* OR
56	“formative evaluation research”
57	S37 OR S38 OR S39 OR S40 OR S41 OR S42 OR S43
58	S49 OR S57

#	Combination of search blocks 1, 2, and 3
59	S29 AND S48 AND S58

For peer review only

Appendix 2. Charting form

CHARTING FORM

ARTICLE NO:

TITLE:

AUTHOR/S:

YEAR OF PUBLICATION:

COUNTRY:

RESEARCH DESIGN: QUANTITATIVE ☐ QUALITATIVE ☐ OTHER: ☐ ________

METHOD:

AIM:

NUMBER OF PARTICIPANTS (n=):

SETTING:

MEDICAL EDUCATION ☐

NURSING EDUCATION ☐

MIDWIFERY EDUCATION ☐

PHYSIOTHERAPY EDUCATION ☐

OCCUPATIONAL THERAPY ☐

DENTAL EDUCATION ☐

OTHER ☐ ________

For peer review only

<u>DOES THE STUDY PRESENT?</u>

HOW PEER ASSESSEMENT INTERVENTIONS ARE DELIVERED	YES	NO	
PEER ASSESSMENT	YES	NO	
FORMATIVE ASSESSMENT	YES	NO	
STUDENTS EXPERIENCE	YES	NO	
TEACHERS EXPERIENCE	YES	NO	OTHER:
OUTCOME/S OF PEER ASSESSMENT INTERVENTION	YES	NO	
RATIONALE/S FOR PEER ASSESSMENT INTERVENTION	YES	NO	

<u>MAIN FINDINGS:</u>

<u>INCLUDED:</u> YES NO

<u>REASON/S FOR EXCLUSION:</u>

For peer review only

(Only to be answered if the study is to be included)

Grading according to the Critical Appraisal Skills Program (CASP)

QUALITATIVE STUDIES AND SYSTEMATIC REVIEWS

LOW ☐ MODERATE ☐ HIGH ☐

RCT AND CASE CONTROL STUDIES

LOW ☐ MODERATE ☐ HIGH ☐

COHORT STUDIES

LOW ☐ MODERATE ☐ HIGH ☐

Grading according to The Mixed Method Appraisal Tool

MIXED METHOD STUDIES

LOW ☐ MODERATE ☐ HIGH ☐

Grading according to the Joanna Briggs Institute Critical Appraisal Tool

QUASI-EXPERIMENTAL STUDIES

LOW ☐ MODERATE ☐ HIGH ☐

ANALYTICAL CROSS SECTIONAL STUDIES

LOW ☐ MODERATE ☐ HIGH ☐

CASE REPORTS

LOW ☐ MODERATE ☐ HIGH ☐

Reviewed by:

(Signature and date)

For peer review only

Preferred Reporting Items for Systematic reviews and Meta-Analyses extension for Scoping Reviews (PRISMA-ScR) Checklist

SECTION	ITEM	PRISMA-ScR CHECKLIST ITEM	REPORTED ON PAGE #
**TITLE**			
Title	1	Identify the report as a scoping review.	1
**ABSTRACT**			
Structured summary	2	Provide a structured summary that includes (as applicable): background, objectives, eligibility criteria, sources of evidence, charting methods, results, and conclusions that relate to the review questions and objectives.	2
**INTRODUCTION**			
Rationale	3	Describe the rationale for the review in the context of what is already known. Explain why the review questions/objectives lend themselves to a scoping review approach.	3, 4
Objectives	4	Provide an explicit statement of the questions and objectives being addressed with reference to their key elements (e.g., population or participants, concepts, and context) or other relevant key elements used to conceptualize the review questions and/or objectives.	5,16
**METHODS**			
Protocol and registration	5	Indicate whether a review protocol exists; state if and where it can be accessed (e.g., a Web address); and if available, provide registration information, including the registration number.	4
Eligibility criteria	6	Specify characteristics of the sources of evidence used as eligibility criteria (e.g., years considered, language, and publication status), and provide a rationale.	5
Information sources*	7	Describe all information sources in the search (e.g., databases with dates of coverage and contact with authors to identify additional sources), as well as the date the most recent search was executed.	5, 23
Search	8	Present the full electronic search strategy for at least 1 database, including any limits used, such that it could be repeated.	23
Selection of sources of evidence†	9	State the process for selecting sources of evidence (i.e., screening and eligibility) included in the scoping review.	5, 6
Data charting process‡	10	Describe the methods of charting data from the included sources of evidence (e.g., calibrated forms or forms that have been tested by the team before their use, and whether data charting was done independently or in duplicate) and any processes for obtaining and confirming data from investigators.	5,6, 25,26,27
Data items	11	List and define all variables for which data were sought and any assumptions and simplifications made.	7,16,22
Critical appraisal of individual sources of evidence§	12	If done, provide a rationale for conducting a critical appraisal of included sources of evidence; describe the methods used and how this information was used in any data synthesis (if appropriate).	6,18,19, 20, 21, 25, 26, 27

For peer review only

SECTION	ITEM	PRISMA-ScR CHECKLIST ITEM	REPORTED ON PAGE #
Synthesis of results	13	Describe the methods of handling and summarizing the data that were charted.	6,7
**RESULTS**			
Selection of sources of evidence	14	Give numbers of sources of evidence screened, assessed for eligibility, and included in the review, with reasons for exclusions at each stage, ideally using a flow diagram.	5,6,17
Characteristics of sources of evidence	15	For each source of evidence, present characteristics for which data were charted and provide the citations.	7,8,9,10
Critical appraisal within sources of evidence	16	If done, present data on critical appraisal of included sources of evidence (see item 12).	18,19,20,21
Results of individual sources of evidence	17	For each included source of evidence, present the relevant data that were charted that relate to the review questions and objectives.	7,8,9,10
Synthesis of results	18	Summarize and/or present the charting results as they relate to the review questions and objectives.	22
**DISCUSSION**			
Summary of evidence	19	Summarize the main results (including an overview of concepts, themes, and types of evidence available), link to the review questions and objectives, and consider the relevance to key groups.	10
Limitations	20	Discuss the limitations of the scoping review process.	11
Conclusions	21	Provide a general interpretation of the results with respect to the review questions and objectives, as well as potential implications and/or next steps.	11
**FUNDING**			
Funding	22	Describe sources of funding for the included sources of evidence, as well as sources of funding for the scoping review. Describe the role of the funders of the scoping review.	12

JBI = Joanna Briggs Institute; PRISMA-ScR = Preferred Reporting Items for Systematic reviews and Meta-Analyses extension for Scoping Reviews.

* Where *sources of evidence* (see second footnote) are compiled from, such as bibliographic databases, social media platforms, and Web sites.

† A more inclusive/heterogeneous term used to account for the different types of evidence or data sources (e.g., quantitative and/or qualitative research, expert opinion, and policy documents) that may be eligible in a scoping review as opposed to only studies. This is not to be confused with *information sources* (see first footnote).

‡ The frameworks by Arksey and O'Malley (6) and Levac and colleagues (7) and the JBI guidance (4, 5) refer to the process of data extraction in a scoping review as data charting.

§ The process of systematically examining research evidence to assess its validity, results, and relevance before using it to inform a decision. This term is used for items 12 and 19 instead of "risk of bias" (which is more applicable to systematic reviews of interventions) to include and acknowledge the various sources of evidence that may be used in a scoping review (e.g., quantitative and/or qualitative research, expert opinion, and policy document).

From: Tricco AC, Lillie E, Zarin W, O'Brien KK, Colquhoun H, Levac D, et al. PRISMA Extension for Scoping Reviews (PRISMA-ScR): Checklist and Explanation. Ann Intern Med. ;169:467–473. doi: 10.7326/M18-0850

For peer review only - http://bmjopen.bmj.com/site/about/guidelines.xhtml

Formative Peer Assessment in Higher Healthcare Education Programs – a Scoping Review

Journal:	*BMJ Open*
Manuscript ID	bmjopen-2020-045345.R1
Article Type:	Original research
Date Submitted by the Author:	25-Nov-2020
Complete List of Authors:	Stenberg, Marie; Malmö Universitet, Care Science<br>Mangrio, Elisabeth; Malmö Universitet, Care Science; Malmö Universitet, Malmö Institute for Studies of Migration, Diversity and Welfare<br>Bengtsson, Mariette; Malmö Universitet, Care Science<br>Carlson, Elisabeth; Malmö Universitet, Care Science
**Primary Subject Heading**:	Medical education and training
Secondary Subject Heading:	Medical education and training
Keywords:	MEDICAL EDUCATION & TRAINING, EDUCATION & TRAINING (see Medical Education & Training), Quality in health care < HEALTH SERVICES ADMINISTRATION & MANAGEMENT

SCHOLARONE™
Manuscripts

I, the Submitting Author has the right to grant and does grant on behalf of all authors of the Work (as defined in the below author licence), an exclusive licence and/or a non-exclusive licence for contributions from authors who are: i) UK Crown employees; ii) where BMJ has agreed a CC-BY licence shall apply, and/or iii) in accordance with the terms applicable for US Federal Government officers or employees acting as part of their official duties; on a worldwide, perpetual, irrevocable, royalty-free basis to BMJ Publishing Group Ltd ("BMJ") its licensees and where the relevant Journal is co-owned by BMJ to the co-owners of the Journal, to publish the Work in this journal and any other BMJ products and to exploit all rights, as set out in our licence.

The Submitting Author accepts and understands that any supply made under these terms is made by BMJ to the Submitting Author unless you are acting as an employee on behalf of your employer or a postgraduate student of an affiliated institution which is paying any applicable article publishing charge ("APC") for Open Access articles. Where the Submitting Author wishes to make the Work available on an Open Access basis (and intends to pay the relevant APC), the terms of reuse of such Open Access shall be governed by a Creative Commons licence – details of these licences and which Creative Commons licence will apply to this Work are set out in our licence referred to above.

Other than as permitted in any relevant BMJ Author's Self Archiving Policies, I confirm this Work has not been accepted for publication elsewhere, is not being considered for publication elsewhere and does not duplicate material already published. I confirm all authors consent to publication of this Work and authorise the granting of this licence.

For peer review only

Formative Peer Assessment in Higher Healthcare Education Programs – a Scoping Review

Marie Stenberg[1] RN; MNSc marie.stenberg@mau.se

Elisabeth Mangrio[1] RN, PhD, Associate Professor elisabeth.mangrio@mau.se

Mariette Bengtsson[1] RN, PhD, Associate Professor mariette.bengtsson@mau.se

Elisabeth Carlson[1] RN, PhD, Professor elisabeth.carlson@mau.se

[1]Department of Care Science, Faculty of Health and Society, Malmö University, SE 205 06 Sweden

Corresponding author: Marie Stenberg, Faculty of Health and Society, Jan Waldenströmsg 25, Malmö University, SE 214 28 Malmö, Sweden

Tel: +46 40 665 79 44

E-Mail: marie.stenberg@mau.se

Word count: 3736

For peer review only

ABSTRACT

Objectives: Formative peer assessment focus learning and development of the student learning process. This implies students taking responsibility for assessing the work of their peers by giving and receiving feedback to each other. The aim was to compile research about formative peer assessment presented in higher healthcare education, focusing the rationale, the interventions, the experiences of students and teachers and the outcomes of formative assessment interventions.

Design: A scoping review.

Data sources: Searches were conducted until May 2019 in PubMed, CINAHL, ERIC, and ERC. Grey literature were searched in Lib-search, Google Scholar and Science Direct.

Eligibility criteria: Studies addressing formative peer assessment in higher education, focusing medicine, nursing, midwifery, dentistry, physical or occupational therapy, and radiology published in peer reviewed articles or in grey literature.

Data extractions and synthesis: Out of 1452 studies, 37 met the inclusion criteria and were critically appraised. The critical appraisal resulted in 18 included studies with high and moderate quality. The pertinent data was analyzed using thematic analysis.

Result: The results present clinical skill-training courses to be a frequent setting for formative peer assessment activities, focusing intra-professional peers. The rationale for using formative peer assessment relates to giving and receiving constructive feedback as a means to promote learning. The experience and outcome of formative peer assessment interventions from the perspective of students and teachers is presented within three themes; 1/organization and structure of the formative peer assessment activities, 2/ personal attributes and consequences for one self and relationships and 3/ experience and outcome of feedback and learning.

Conclusion: Healthcare education must consider preparing and introducing students to collaborative learning and thus develop of well-designed learning activities aligned with the learning outcomes. Since peer collaboration seems to affect students' and teachers' experiences of formative peer assessment empirical investigations exploring collaboration between students is of utmost importance.

Keywords: Feedback, formative assessment, healthcare education, peer assessment, students, teachers

For peer review only

For peer review only - http://bmjopen.bmj.com/site/about/guidelines.xhtml

STRENGTHS AND LIMITATIONS OF THE STUDY

- The current scoping review is previously presented in a published study protocol.
- Four databases were systematically searched to identify research on formative peer assessment.
- Critical appraisal tools were used to assess the quality of studies with quantitative, qualitative, and mixed methods designs.
- Articles appraised with high or moderate quality were included.
- Since only English studies were included, studies may have been missed that would otherwise have met the inclusion criteria.

BACKGROUND

Peer assessment is an educational approach where feedback, communication, reflection, and collaboration between peers are key characteristics. In a peer assessment activity, students take responsibility for assessing the work of their peers by giving (and receiving) feedback on a specific subject.[1] It allows students to consider the learning outcomes for peers of similar status and to reflect upon their own learning mirrored in a peer.[2] Peer assessment has shown to support students' development of judgement skills, critiquing abilities, and self-awareness as well as their understanding of the assessment criteria used in a course.[1] In higher education, peer assessment has been a way to move from an individualistic and teacher-led approach to a more collaborative, student-centred approach to assessment[1] aligned with social constructivism principles.[3] In this social context of interaction and collaboration, students can expand their knowledge, identify their strengths and weaknesses, and develop personal and professional skills[4] by evaluating the professional competence of a peer.[5] Peer assessment can be used in academic and professional settings as a strategy to enhance students' engagement in their own learning.[6 7 8] The collaborative aspect of peer assessment relates to professional teamwork, as well as to broader goals for lifelong learning. As argued by Boud et al.,[1] peer assessment addresses course-specific goals not readily developed otherwise. For healthcare professions, it enhances ability to work in a team in a supportive and respectful atmosphere,[9] which is highly relevant for patient outcome and the reduction of errors for patient safety.[10]

For peer review only - http://bmjopen.bmj.com/site/about/guidelines.xhtml

However, recent research has shown that peer collaboration is challenging[11] and that healthcare professionals are not prepared to deliver and receive feedback effectively.[12] This emphasizes the importance for healthcare educators to support students with activities fostering these competences. Feedback is highly associated with enhancing student learning[13] and modifying learning during the learning process[14] so students can close the gap between their present state of learning and their desired goal(s). Peer feedback can be written or oral, conducted as peer observations in small or large groups.[8] Further, it is driven by set assessment criteria,[1] which can be either summative or formative, formal or informal. Summative assessment evaluates students' success or failure after the learning process,[15] whereas formative assessment aims for improvement during the learning process.[16,4] According to Black and Wiliam,[15] formative peer assessment activities involve feedback to modify the teaching and learning of the students. The intention of feedback is to help students help each other when planning their learning.[4 17] An informal formative peer assessment activity involves a continuous process throughout a course or education, whereas a formal one is designated to a single point in a course's momentum. Earlier research on peer assessment in healthcare education has provided an overview of specific areas within the peer assessment process. For example, Speyer, Pilz, and Van Der Kruis presented psychometric characteristics of peer assessment instruments and questionnaires in medical education,[18] concluding that quite a few instruments exist; however, these intruments mainly focus on professional behavior, and they lack sufficient psychometric data. Tornwall[12] focused on how nursing students are prepared by academics to participate in peer assessment activities and highlighted the importance of creating a supporting learning environment. Lerchenfeldt, Mi and Eng[19] concluded that peer assessment supports medical students in developing professional behavior and that peer feedback is a way to assess professionalism. Khan, Payne, and Chahine[20] reviewed the role of peer assessment in objective structured clinical examination (OSCE), showing that peer assessment promotes learning but that students need training in how to provide feedback. In short, the existing literature contributes valuable knowledge about formative peer assessment in healthcare education targeting specific areas. However, there seems to be lack of compiled research considering formative peer assessment in its entirety, including the context, rationale, experience, and outcome of the formative peer assessment process. Therefore, this scoping review attempts to present an overview of formative peer assessment in healthcare education rather than specific areas within that process.

METHOD

For peer review only - http://bmjopen.bmj.com/site/about/guidelines.xhtml

This scoping review was conducted using the York methodology by Arksey and O'Malley [21] and the recommendations presented by Levac, Colquhuan, and O'Brien.[22] We constructed a scoping protocol, utilizing a PRISMA-P protocol, to present the planned methodology for the scoping review.[23]

Aim and research questions

We aimed to compile research about formative peer assessment presented in higher healthcare education. The research questions were as follows: What are the rationales for using formative peer assessment in healthcare education? How are formative peer assessment interventions delivered in healthcare education and in what context? What experiences of formative peer assessment do students and teachers in healthcare education have? What are the outcomes of formative peer assessment interventions? We used the "Population Concept and Context" (PCC) elements recommended for scoping reviews to establish effective search criteria (Table 1).[24]

INSERT TABLE 1 HERE

Relevant studies identified

The literature search was conducted in the databases PubMed, Cumulative Index to Nursing and Allied Health Literature (CINAHL), Education Research Complete (ERIC), and Education Research Centre (ERC). Search tools such as Medical Subject Headings, Headings, Thesaurus and Boolean operators (AND/OR) helped expand and narrow the search. Initially, the search terms were broad (e.g., peer assessment or higher education) in order to capture the range of published literature. However, the extensiveness of material made it necessary to narrow the search terms and organize them in three major blocks. The following inclusion criteria were applied in the search: (a) articles addressing formative peer assessment in higher education; (b) students and teachers in medicine, nursing, midwifery, dentistry, physical or occupational therapy, and radiology; and (c) peer reviewed articles, grey literature (books, discussion papers, posters et cetera). Studies of summative peer assessment, instrument development, and systematic reviews were excluded. We incorporated several similar terms related to peer assessment in the search to ensure that no studies were missed (Appendix 1). Furthermore, we consulted a well-versed librarian with experience of systematic search[25] to assist us in systematically identifying relevant databases and search terms for each database, control the relevance of the constructed search blocks, and manage the data in a reference

For peer review only - http://bmjopen.bmj.com/site/about/guidelines.xhtml

management system. No limitation were set for year, all studies indexed in the four databases were included until the last search in May 28, 2019.

Study selection

The process of the study selection and the reasons for exclusion are presented in a flow diagram[26] (Figure 1). First, the first author (MS) screened all 1,452 titles. Second, MS read all the abstracts, gave those responding to the research questions a unique code, and organized them in a reference management system. The reason for inclusion and exclusion at title and abstract level was charted by the first author and critically discussed within the team (MS, EM, MB, EC). An additional hand search of reference lists was conducted. To cover a subject in full, a scoping review should include search in grey literature.[21 22] Therefore, the grey literature was scoped to find unpublished results by searching Google Scholar, LibSearch, and Science Direct. The grey literature mostly contained research posters, conference abstracts, discussion papers, and books, but a hand search revealed original research articles that were added for further screening and appraisal. Finally, the first author (MS) arrived at 81 studies, read them in full-text, and discussed them with the other three authors (EM, MB, EC).

INSERT FIGURE 1 HERE

Charting the data

We constructed a charting form to facilitate the screening of the full-text studies (Appendix 2). Out of the 81 studies, 37 met the inclusion criteria and were appraised for quality using Critical Appraisal Skills Programme (CASP).[27] The reason for conducting a crtitical appraisal of the studies was to enhance the use of the findings for policy-making and practice in higher healthcare education.[28] To investigate the interpretation of the quality instrument, three members of the research team (MS, EM, EC) conducted an initial test assessment of two randomly selected studies and graded them with high, moderate, or low quality. Additional screening tools were used for studies with a mixed methods design[29] and cross-sectional studies[30] not available in CASP. When a discrepancy arose, a fourth researcher (MB) assessed the articles independently without prior knowledge of what the others have concluded. This was followed by a discussion among all four researchers to secure internal agreement on how to further interpret the checklist items and quality assessment. Consequently, to ensure high quality, the studies had to have a “yes” answer for a majority of the questions. If “no” dominated, the study was excluded. Since earlier reports[31] have raised and discussed the importance of ethical issues in systematic reviews, all screening protocols in this review

For peer review only

included ethical considerations, as an individual criterion. The first author critically appraised all 37 articles, and 15 articles were divided between the team members (EM, MB, EC) and independently appraised. Nevertheless, during the screening process all 37 articles were critically discussed using the Rayyan system for systematic reviews[32] before final descision of inclusion. By this procedure, all authors were in agreement of not only which articles to include, but also the reason for exclusion. The critical appraisal resulted in 18 studies with high and moderate quality (Table 2).

INSERT TABLE 2 HERE

Collating, summarizing, and reporting results

The analysis process followed the five phases of thematic analysis described by Braun and Clarke,[33] with support of a practical guide provided by Maquire & Delahunt.[34] The first phase includeed familiarizing with the data. Therefore, prior to the coding process, we read all the articles to grasp a first impression of the results presented within the included studies.We then conducted a theoretical thematic analysis, meaning that the results were deductively coded,[33] guided by the research questions. We read the results a second time before starting the initial coding. The codes consisted of short descriptions close to the original text. The codes were then combined into themes and sub-themes. The themes were identified with a semantic approach, meaning that they were explicit: we did not look for anything beyond what was written.[33] Finally, we constructed a thematic map to present an overview of the results and how they related to each other. The results from the studies are presented narratively.

Consultation

Consultation is an optional stage in scoping reviews.[21] However, since it adds methodologic rigor,[22] we presented and discussed the preliminary results and the thematic map with nine academic teachers, experts within the field of healthcare education and pedagogy. The purpose of the consultation was to enhance the validity of the results of the scoping review and to facilitate appropriate dissemination of outputs.[33] The expert group responded to four questions: Do the themes make sense? Is too much data included in one single theme? Are the themes distinct or do they overlap? Are there themes within themes?[34] The consultation resulted in a revision of a few themes and the way they related to each other.

RESULTS

For peer review only - http://bmjopen.bmj.com/site/about/guidelines.xhtml

The 18 included studies were published between 2002 and 2017 in the United States (6), the United Kingdom (6), Australia (3), Canada (2), and the United Arab Emirate (1) (Table 3). The studies were conducted in medical (12), dental (2), nursing (2), occupational therapy (1), and radiography (1) educations. Six studies were presented in the framework of an existing collaborative educational model.[35-37 38 39 40] Our review revealed that the most frequent setting for formative peer assessment activities is within clinical skill-training courses,[35 39-47] involving intra-professional peers. The common rationale for using formative peer assessment is to support students, usually explained by the inherent learning of the feedback process,[35 39 40 43-45 47-51] and to prepare students for professional behavior and provide them with the skills required in the health care professions.[36 37 38 46 47 48 49 52] Table 3 presents the results of the analysis related to the research questions of context, rationale, and interventions of formative peer assessment.

INSERT TABLE 3 HERE

The results related to the research questions about the experience of students and teachers and the outcome of formative peer assessment interventions fall within three themes: (1) the organization and structure of peer assessment activities, (2) personal attributes and consequences for oneself and one's peer relationships, and (3) the experience and outcome of feedback and learning.

The organization and structure of formative peer assessment activities

In the reviewed studies, students express viewing the responsibility of faculty as a key component in formative peer assessment, meaning that faculty must clearly state the aim of the peer assessment activity. Students highlight the need to be prepared and trained in how to give and receive constructive feedback.[36 47 50-52] The learning activities need to be well-designed and supported by guidelines on how to use them.[35 36 50 52] Otherwise, it could discourage students from participating in the peer activities.[52] Novice students find it difficult to be objective and to offer constructive criticism in a group.[36 46] This emphasizes the importance of responsibility from faculty, especially when students are to give feedback on professional behavior.[52] Some students prefer direct communication with peers when feedback is negative, whereas others think it is the responsibility of faculty.[52] There is some ambiguity regarding whether feedback should be given anonymously or not,[47 52] whether it should bear consequences from faculty or not,[52]

For peer review only - http://bmjopen.bmj.com/site/about/guidelines.xhtml

whether it should be informal or formal, and whether the peer should be at the same academic level or at a more experienced higher-level.[50 52] However, some students express favoring small groups;[41 49] further, students in small groups show more activity than those in large groups.[41] Students and teachers agree that peer assessment should be strictly formative rather than summative.[42 46 52] Teachers see themselves as key facilitators and express that students value feedback from teachers rather than from peers (in terms of credibility).[51] Students express similar sentiments even if they appreciated the peer feedback.[40 42 44 46] However, teachers confirm the need for training and preparing students early in the education, as well as the need for their own professional development to guide students effectively.[51]

Personal attributes and the impact and consequences for oneself and one's peer relationships

Students generally focus on how peer assessment activities may affect their personal relationships in a negative way.[35 37 42 50 52] They express worry over consequences for themselves and their social relationships[37 40 52] as well as anxiety that negative feedback given to a peer may affect the grading from faculty.[52] Moreover, students emphasize the importance of enthusiasm and engagement in listening to peers' opinions during their collaboration.[36 47] They mention positive personal attributes and behaviors such as being organized, polite, and helpful as a support for peer collaboration.[36 47] Further, they mention the importance of both a positive and close relationship between students and faculty[52] and a positive culture in the learning environment.[40] While students highlight the impact and consequences on personal relationships, teachers speak of the importance of respect in formative peer assessment,[36] including respect for each other, the learning activity, and the collaboration and interaction.[36] Further, teachers emphasize the importance of students being self-aware, being well prepared, and taking own responsibility for the peer assessment activity.[36]

The experience and outcome of feedback and learning

According to the students in the reviewed studies, formative peer assessment contributes to developing the skills needed in practice and in their future profession.[35 36 40 41 48 52] They appreciate the opportunity to give and receive feedback from a peer,[35 36 40 42 47 48 50] and they agree that the feedback they received made them change how they worked[42 48] or how they taught their peers.[47 48] They consider activities such as observation of others'

For peer review only - http://bmjopen.bmj.com/site/about/guidelines.xhtml

performance as beneficial for learning because they make them reflect on their own performance[35 36 40 41 46 49 50] and help them identify knowledge gaps.[35 40 49] Students with prior experience of peer learning are more likely to provide specific guiding feedback than those without such experiences.[39] Moreover, two studies showed significantly improved test results for students who took part in a peer feedback activity compared to those who did not.[43 49] Further, students think they could be honest in their feedback and would learn better if the feedback was more in-depth.[35 46] Students at entry level tend to give more positive feedback than senior students; they also focus on practical and clinical knowledge, whereas students in year five focus on communication, management, and leadership in their feedback comments.[45] A study exploring what students remember of received feedback shows they remember positive growth, negative self-image, and negative attitudes toward classmates. Received feedback sometimes confirmed personal traits the students already knew about.[37] In addition, negative feedback was more likely to result in a change in their work habits and interpersonal attributes.[37] Students express some anxiety regarding the usefulness of feedback from low-performing students[40 50] and non-motivated students, which contributes to ineffective interaction and learning.[36 47] Low performing students showlack of initiative, preparation, and respect but also show improvement in their ratings after the peer assessment experience.[47] Furthermore, feedback from peers can be a predictor of a student's unprofessional behavior; hence, it could be used as a tool for early remediation.[38] In an evaluation of faculty examiners' experience of students' feedback, the faculty express they view student feedback to be given in a professional and appropriate way and faculty examiners would have given similar feedback.[42] In an OSCE-examination where a checklist was used, the results show statistical significance in assessment between faculty examiners and student examiners.[42]

DISCUSSION

We conclude that formative peer assessment is a process with two consecutive phases. The first phase concerns the understanding of the rationale and fundament of the peer assessment process for students and faculty members. The results indicate that the rationale is to support student learning and prepare them for healthcare professions. The formative peer assessment activities support students' reflection on their own knowledge and development when mirrored in a peer by altinating the role as being both observed and observer..[53 54] It further contributes to skills as communication, transfer of understandable knowledge and collaboration, all significant core competences when caring for patients and their relatives.[54]

For peer review only - http://bmjopen.bmj.com/site/about/guidelines.xhtml

For faculty, organizing formative peer assessment, can be cost beneficial. This was recently emphasized in high volume classes expressing the reduction of costs with students giving feedback to a peer instead of teachers.[55] Nevertheless, students express the importance of clarifying the aim of the peer assessment activity and the responsibility of the faculty. We recommend faculty to clearly define the activity and explain how it supports student learning and professionalism, especially when students are to provide feedback to each other on sensitive matters, such as un-professional behavior. A collaborative activity requires trust, and the real intention must be made transparent. [4 56-58] Moreover, to enable student development in-line with the learning outcomes, the learning activity needs to be well designed and understood by students so they can advantageously relate to the purpose.[59 60 61] However, Casey et al.[62] recommended further investigations of how to prepare students for the peer assessment activities.

The second phase concerns the organization and structure of the formative peer assessment activity, for example, how to give and receive feedback. The current scoping review reveals the complexity of peer collaboration in formative peer assessment: It affects students' emotions concerning both themselves and their relationship with their peers. This coincides with earlier research emphasizing the social factors of peer assessment and the importance for teachers to consider them.[4] Nevertheless, surprisingly, few studies highlight the collaborative part of peer assessment.[4 11] One reason might be that formative peer assessment is often presented as a "stand alone" activity and not involved in a collaborative learning environment.[8 63] We agree with earlier research[64 65] arguing that peer assessment needs to be affiliated with practices of collaborative learning. Similar implications are presented by Tornwall,[12] who concluded the importance of integrating peer collaboration as a natural approach throughout education to support student development.

CONCLUSIONS AND IMPLICATIONS FOR FURTHER RESEARCH

Some have argued that the research on peer assessment is deficient in referring to exactly what peer assessment aims to achieve.[66] We conclude that within healthcare education the aim of formative peer assessment is to prepare students for the collaborative aspects crucial within the healthcare professions. However, healthcare education must consider preparing and introducing students to collaborative learning; therefore, it must develop well-designed learning activities aligned with the learning outcomes. Based on this scoping review,

For peer review only - http://bmjopen.bmj.com/site/about/guidelines.xhtml

formative peer assessment needs to be implemented in a collaborative learning environment throughout the education to be effective. However, since peer collaboration seems to affect students' and teachers' experiences of formative peer assessment, empirical investigations exploring the collaboration between students are of utmost importance.

LIMITATIONS

Previous methodological concerns and discussions have been related to the systematic approach of handling grey literature.[67 68] We argue that the grey literature contributes to a wider understanding of the research area. When we were conducting a critical appraisal of included studies, the grey literature was excluded due to lack of methodological rigor. Therefore, we recommend considering this time-consuming phase of the methodology in scoping reviews. We further acknowledge that the last search was conducted in May 2019 and studies may have been included if an additional search had been provided after this date and in other databases than the ones presented. Further, the current scoping review has not fully elucidated the perspective of teachers and faculty. Few of the included studies highlight the teachers' perspective, so further research is required.

Authors' contribution: MS led the design, search strategy, and conceptualization of this work and drafted the manuscript. EM, MB, and EC were involved in the conceptualization of the review design, inclusion and exclusion criteria, and critical appraisal and provided feedback on the methodology and the manuscript. All authors give their approval to the publishing of this scoping review manuscript.

Funding statement: This research received no specific grant from any funding agency in the public, commercial, or not-for-profit sectors.

Competing interests: None declared.

Patient and public involvement: As this is a scoping review no primary research has been conducted and thus not involve patients or the public.

Acknowledgement: Special thanks to the members in the expert group for their valuable contribution in the consultation.

Additional data: No additional data available.

Figure 1. PRISMA Flowchart

REFERENCES

For peer review only

For peer review only - http://bmjopen.bmj.com/site/about/guidelines.xhtml

1. Boud D, Cohen R, Sampson J. Peer learning and assessment. *Assessment & evaluation in higher education* 1999;24(4):413-26.
2. Topping KJ, Ehly SW. Peer assisted learning: A framework for consultation. *Journal of Educational and Psychological Consultation* 2001;12(2):113-32.
3. Vygotskiĭ LS. Thought and language: MIT press 2012.
4. Topping KJ. Peer Assessment. 2009:20-27. doi: 10.1080/00405840802577569
5. Dannefer EF, Henson LC, Bierer SB, et al. Peer assessment of professional competence. *Medical Education* 2005;39(7):713-22. doi: 10.1111/j.1365-2929.2005.02193.x
6. Casey D, Burke E, Houghton C, et al. Use of peer assessment as a student engagement strategy in nurse education. *Nursing & health sciences* 2011;13(4):514-20.
7. Orsmond* P, Merry S, Callaghan A. Implementation of a formative assessment model incorporating peer and self-assessment. *Innovations in Education and Teaching International* 2004;41(3):273-90.
8. Ashenafi MM. Peer-assessment in higher education – twenty-first century practices, challenges and the way forward. *Assessment and Evaluation in Higher Education* 2017;42(2):226-51. doi: 10.1080/02602938.2015.1100711
9. Sims S, Hewitt G, Harris R. Evidence of collaboration, pooling of resources, learning and role blurring in interprofessional healthcare teams: a realist synthesis. *Journal of Interprofessional care* 2015;29(1):20-25.
10. Morley L, Cashell A. Collaboration in health care. *Journal of medical imaging and radiation sciences* 2017;48(2):207-16.
11. Stenberg M, Carlson E. Swedish student nurses' perception of peer learning as an educational model during clinical practice in a hospital setting—an evaluation study. *BMC nursing* 2015;14(1):48.
12. Tornwall J. Peer assessment practices in nurse education: An integrative review, 2018:266-75.
13. Nicol DJ, Macfarlane-Dick D. Formative assessment and self-regulated learning: A model and seven principles of good feedback practice. *Studies in higher education* 2006;31(2):199-218.
14. Hudson JN, Bristow DR. Formative assessment can be fun as well as educational. *Advances in physiology education* 2006;30(1):33-37. doi: 10.1152/advan.00040.2005
15. Black P, Wiliam D. Assessment and classroom learning. *Assessment in Education: principles, policy & practice* 1998;5(1):7-74.
16. Sadler DR. Beyond feedback: Developing student capability in complex appraisal. *Assessment & Evaluation in Higher Education* 2010;35(5):535-50.
17. Topping K. Peer assessment between students in colleges and universities. *Review of educational Research* 1998;68(3):249-76.
18. Speyer R, Pilz W, Van Der Kruis J, et al. Reliability and validity of student peer assessment in medical education: a systematic review. *Med Teach* 2011;33(11):e572-85. doi: 10.3109/0142159X.2011.610835 [published Online First: 2011/10/26]
19. Lerchenfeldt S, Mi M, Eng M. The utilization of peer feedback during collaborative learning in undergraduate medical education: a systematic review. *BMC medical education* 2019;19(1):321.
20. Khan R, Payne MWC, Chahine S. Peer assessment in the objective structured clinical examination: A scoping review. *Medical Teacher* 2017;39(7):745-56. doi: 10.1080/0142159X.2017.1309375
21. Arksey H, O'Malley L. Scoping studies: towards a methodological framework. *International Journal of Social Research Methodology* 2005;8(1):19-32. doi: 10.1080/1364557032000119616
22. Levac D, Colquhoun H, O'Brien KK. Scoping studies: advancing the methodology. *Implementation Science* 2010;5(1):69. doi: 10.1186/1748-5908-5-69
23. Stenberg M, Mangrio E, Bengtsson M, et al. Formative peer assessment in healthcare education programmes: protocol for a scoping review. *BMJ open* 2018;8(11):e025055-e55. doi: 10.1136/bmjopen-2018-025055

For peer review only

For peer review only - http://bmjopen.bmj.com/site/about/guidelines.xhtml

24. Peters M, Godfrey C, McInerney P, et al. Methodology for JBI scoping reviews. The Joanna Briggs Institute Reviewers manual 2015: The Joanna Briggs Institute 2015:3-24.
25. Morris M, Boruff JT, Gore GC. Scoping reviews: establishing the role of the librarian. *Journal of the Medical Library Association: JMLA* 2016;104(4):346.
26. Moher D, Liberati A, Tetzlaff J, et al. Preferred reporting items for systematic reviews and meta-analyses: the PRISMA statement. *Annals of internal medicine* 2009;151(4):264-69.
27. Programme CAS. Critical Appraisal Skills Programme. CASP checklist 2018 [Available from: https://casp-uk.net/casp-tools-checklists/ accessed 5 august 2018.
28. Daudt HM, van Mossel C, Scott SJ. Enhancing the scoping study methodology: a large, inter-professional team's experience with Arksey and O'Malley's framework. *BMC medical research methodology* 2013;13(1):48.
29. Hong Q, Pluye P, Fàbregues S, et al. Mixed Methods Appraisal Tool (MMAT) version 2018: User guide. *Department of Family Medicine, McGuill Univertiy* 2018
30. Institute JB. Critical Appraisal Tools [Available from: http://joannabriggs.org/research/critical-appraisal-tools.html accessed 5 august 2018.
31. Weingarten MA, Paul M, Leibovici L. Assessing ethics of trials in systematic reviews. *Bmj* 2004;328(7446):1013-14.
32. Ouzzani M, Hammady H, Fedorowicz Z, et al. Rayyan—a web and mobile app for systematic reviews. *Systematic reviews* 2016;5(1):210.
33. Braun V, Clarke V. Using thematic analysis in psychology. *Qualitative research in psychology* 2006;3(2):77-101.
34. Maguire M, Delahunt B. Doing a thematic analysis: A practical, step-by-step guide for learning and teaching scholars. *AISHE-J: The All Ireland Journal of Teaching and Learning in Higher Education* 2017;9(3)
35. Cushing A, Abbott S, Lothian D, et al. Peer feedback as an aid to learning - What do we want? Feedback. When do we want it? Now! *Medical Teacher* 2011;33(2) doi: 10.3109/0142159X.2011.542522
36. Iqbal M, Velan GM, O'Sullivan AJ, et al. Differential impact of student behaviours on group interaction and collaborative learning: medical students' and tutors' perspectives. *BMC medical education* 2016;16(1):217-17. doi: 10.1186/s12909-016-0730-1
37. Nofziger AC, Naumburg EH, Davis BJ, et al. Impact of peer assessment on the professional development of medical students: a qualitative study. *Academic medicine : journal of the Association of American Medical Colleges* 2010;85(1):140-47. doi: 10.1097/ACM.0b013e3181c47a5b
38. Emke AR, Cheng S, Chen L, et al. A Novel Approach to Assessing Professionalism in Preclinical Medical Students Using Multisource Feedback Through Paired Self- and Peer Evaluations. *Teaching and Learning in Medicine* 2017;29(4):402-10. doi: 10.1080/10401334.2017.1306446
39. Chou CL, Masters DE, Chang A, et al. Effects of longitudinal small-group learning on delivery and receipt of communication skills feedback. *Medical Education* 2013;47(11):1073-79. doi: 10.1111/medu.12246
40. Tai JH-M, Canny BJ, Haines TP, et al. The role of peer-assisted learning in building evaluative judgement: opportunities in clinical medical education. *Advances in health sciences education : theory and practice* 2016;21(3):659-76. doi: 10.1007/s10459-015-9659-0
41. Cho Y, Je S, Yoon YS, et al. The effect of peer-group size on the delivery of feedback in basic life support refresher training: A cluster randomized controlled trial. *BMC Medical Education* 2016;16(1):1-8. doi: 10.1186/s12909-016-0682-5
42. Moineau G, Power B, Pion A-MJ, et al. Comparison of student examiner to faculty examiner scoring and feedback in an OSCE. *Medical education* 2011;45(2):183-91. doi: 10.1111/j.1365-2923.2010.03800.x
43. Satterthwaite JD, Grey NJA. Peer-group assessment of pre-clinical operative skills in restorative dentistry and comparison with experienced assessors. *European Journal of Dental Education* 2008;12(2):99-102. doi: 10.1111/j.1600-0579.2008.00509.x

For peer review only

For peer review only - http://bmjopen.bmj.com/site/about/guidelines.xhtml

44. Vaughn CJ, Kim E, O'Sullivan P, et al. Peer video review and feedback improve performance in basic surgical skills. *American Journal of Surgery* 2016;211(2):355-60. doi: 10.1016/j.amjsurg.2015.08.034
45. Tricio J, Woolford M, Escudier M. Analysis of dental students' written peer feedback from a prospective peer assessment protocol. *European Journal of Dental Education* 2016;20(4):241-47.
46. Rees C, Sheard C, McPherson A. Communication skills assessment: the perceptions of medical students at the University of Nottingham. *Medical Education* 2002;36(9):868-78.
47. Spandorfer J, Puklus T, Rose V, et al. Peer assessment among first year medical students in anatomy. *Anatomical sciences education* 2014;7(2):144-52. doi: 10.1002/ase.1394
48. Martin D, Friesen E, De Pau A. Three heads are better than one: a mixed methods study examining collaborative versus traditional test-taking with nursing students. *Nurse education today* 2014;34(6):971-77. doi: 10.1016/j.nedt.2014.01.004
49. Mui Lim S, Rodger S. The use of interactive formative assessments with first-year occupational therapy students. *International Journal of Therapy and Rehabilitation* 2010;17(11):576-86.
50. Elshami W, Abdalla ME. Diagnostic radiography students' perceptions of formative peer assessment within a radiographic technique module. *Radiography (London, England : 1995)* 2017;23(1):9-13. doi: 10.1016/j.radi.2016.06.001
51. Koh LC. Academic staff perspectives of formative assessment in nurse education. *Nurse education in practice* 2010;10(4):205-09. doi: 10.1016/j.nepr.2009.08.007
52. Arnold L, Shue CK, Kritt B, et al. Medical students' views on peer assessment of professionalism. *Journal of General Internal Medicine* 2005;20(9):819-24. doi: 10.1111/j.1525-1497.2005.0162.x
53. Carlson E, Stenberg M, Chan B, et al. Nursing as universal and recognisable: Nursing students' perceptions of learning outcomes from intercultural peer learning webinars: A qualitative study. *Nurse education today* 2017;57:54-59.
54. Homberg A, Hundertmark J, Krause J, et al. Promoting medical competencies through a didactic tutor qualification programme–a qualitative study based on the CanMEDS Physician Competency Framework. *BMC Medical Education* 2019;19(1):1-8.
55. Schwill S, Fahrbach-Veeser J, Moeltner A, et al. Peers as OSCE assessors for junior medical students–a review of routine use: a mixed methods study. *BMC medical education* 2020;20(1):1-12.
56. Vickerman P. Student perspectives on formative peer assessment : an attempt to deepen learning ? 2009;34(2):221-30. doi: 10.1080/02602930801955986
57. Sluijsmans D, Prins F. A conceptual framework for integrating peer assessment in teacher education. *Studies in Educational Evaluation* 2006;32(1):6-22.
58. Boud D, Falchikov N. Developing assessment for informing judgement. *Rethinking assessment in higher education: Learning for the longer term* 2007:181-97.
59. Cassidy S. Developing employability skills : peer assessment in higher education. 2006 doi: 10.1108/00400910610705890
60. Solheim E, Plathe HS, Eide H. Nursing students' evaluation of a new feedback and reflection tool for use in high-fidelity simulation – Formative assessment of clinical skills. A descriptive quantitative research design. *Nurse Education in Practice* 2017;27:114-20. doi: 10.1016/j.nepr.2017.08.021
61. Topping KJ. Peer assessment. *Theory into practice* 2009;48(1):20-27.
62. Casey D, Burke E, Houghton C, et al. Use of peer assessment as a student engagement strategy in nurse education. *Nursing and Health Sciences* 2011;13(4):514-20. doi: 10.1111/j.1442-2018.2011.00637.x
63. Papinczak T, Young L, Groves M, et al. An analysis of peer, self, and tutor assessment in problem-based learning tutorials. *Med Teach* 2007;29(5):e122-32. doi: 10.1080/01421590701294323 [published Online First: 2007/09/22]

For peer review only - http://bmjopen.bmj.com/site/about/guidelines.xhtml

64. Kollar I, Fischer F. Peer assessment as collaborative learning: A cognitive perspective. *Learning and Instruction* 2010;20(4):344-48.
65. Finn GM, Garner J. Twelve tips for implementing a successful peer assessment. *Medical teacher* 2011;33(6):443-46.
66. Jackel B, Pearce J, Radloff A, et al. Assessment and feedback in higher education.
67. Gentles SJ, Lokker C, McKibbon KA. Health information technology to facilitate communication involving health care providers, caregivers, and pediatric patients: a scoping review. *Journal of medical Internet research* 2010;12(2):e22.
68. Pham MT, Rajić A, Greig JD, et al. A scoping review of scoping reviews: advancing the approach and enhancing the consistency. *Research synthesis methods* 2014;5(4):371-85.

For peer review only

For peer review only - http://bmjopen.bmj.com/site/about/guidelines.xhtml

Table 1. The PCC mnemonic as recommended by the Joanna Briggs Institute

Population	Concept	Context
Students assessing students	Intervention, rationale, outcome, context, and students' and teachers' experience of formative peer assessment	Healthcare education programs in higher education

For peer review only - http://bmjopen.bmj.com/site/about/guidelines.xhtml

Table 2. Overview of included studies.

Author, year, country, and journal	Aim	Design	Participants	Main findings	Quality*
Arnold et al., 2005,[52] USA<br><br>*Journal of General Internal Medicine*	Identify factors that encourage or discourage student participation in peer assessment	Qualitative Grounded theory<br><br>Focus groups (16) at two medical schools	n=61, medical students in year 1, 3, and 4	The characteristics of the peer assessment system and the environment can encourage or discourage participation. Themes: (1) Students' struggle with peer assessment, (2) Characteristics of a peer assessment system, and (3) Environmental factors	moderate
Cho et al., 2016,[41] England<br><br>*BMC Medical Education*	Investigate the effect of peer-group size on competency-based skills	Quantitative Cluster RCT	n=115, medical students in year 6	Smaller groups (4.1) show more active and preferred than large groups (8,1). Group size did not impact scores.	high
Chou et al., 2013,[39] USA<br><br>*Medical Education*	Examine the role of prior peer-learning relationships between students in their delivery and receipt of feedback on clinical communication skills	Mixed method Case-control Descriptive statistics Survey, video observations	n= 72 medical students in year 3 with prior peer learning relationships<br><br>n=36 students in control group with no prior peer relationships.	Students with prior peer learning relationships more likely to provide specific corrective feedback than those without prior relationships. No significant difference between groups regarding how feedback was received.	moderate
Cushing et al., 2011,[35] United Kingdom<br><br>*Medical Teacher*	Investigate the benefits of formative peer feedback in communication skills and develop a training programme in peer feedback	Mixed method Questionnaire (20 items) at two occasions with 6 months in between. Focus groups (5 medical- and 2 nurse students)	n=45 medical students in year 1<br><br>n=48 nursing students in year 1	Students valued the learning opportunity of both being examiner and observer. They preferred more in-depth feedback and feedback from tutors. They expressed anxiety about giving negative feedback to a peer and had mixed views on giving feedback (relaxed or pressured) and its use in clinical placements.	high
Elshami & Abdalla, 2017,[50] United Arab Emirates<br><br>*Radiography*	Assess perception of formative peer assessment	Qualitative Action research Focus groups (3) Content analysis	n=19 (24**) diagnostic radiography students in year 3	Formative peer assessment gives valuable feedback from same level or more experienced peers. Need for training and detailed rubrics.	moderate
Emke et al., 2017,[38] USA	Demonstrate that perceptual errors related to professionalism behaviors can be detected early through	Quantitative	n=246 medical students in year 2	Multiple peer assessments and feedback a tool predictor of unprofessional behavior.	moderate

For peer review only - http://bmjopen.bmj.com/site/about/guidelines.xhtml

Teaching and Learning in Medicine	repeated multisource feedback				
Iqbal et al., 2016,[36]<br>Australia<br><br>*BMC Medical Education*	Explore students' and tutors' perception of key collaborative behaviors that impact collaborative learning and interaction	Qualitative<br>Focus groups (5) with students<br>Interviews (8) with teachers<br>Thematic analysis	n= 22 medical students in year one and two<br><br>n= 8 teachers	Being respectful, giving constructive feedback, and being engaged and prepared had positive impact on both learning and group interaction. Passiveness, unreliability, irresponsibility, and condescending attitudes had a negative impact on learning and interaction. Similar results from teachers.	high
Koh, 2010,[51]<br>United Kingdom<br><br>*Nurse Education in Practice*	Explore how academic staff experience, understand, and interpret the process of formative assessment and feedback of theoretical assessment	Qualitative<br>Phenomenology<br>Semi-structured interviews (22)<br>Thematic analysis	n=20 academic staff in nurse education	Teachers see themselves as key facilitators and think students prefer teacher feedback. Students are assumed to have the skill to peer assess and give feedback but are unprepared and need support and introduction early in education. Teachers need professional development themselves.	moderate
Mui Lim & Rodger, 2010.[49]<br>Australia<br><br>*International Journal of Therapy and Rehabilitation*	Improve students learning through interactive formative assessment and student generated questions	Mixed methods<br>Cohort study<br>Evaluation questionnaire	n= 115 occupational therapy students in year 1 in 2009 compared with n= 98 students in 2008	Significant improvement in exams result from being part of interactive formative assessment, which is beneficial for learning and identifying knowledge gaps.	moderate
Martin, Friesen, & De Pau, 2014,[48]<br>Canada<br><br>*Nurse Education Today*	Examine collaborative testing versus traditional test taking with undergraduate nursing students in a nine-station OSCE	Mixed method<br>Cross over design<br>Survey<br>Focus groups	n=70 nursing students	Significantly higher scores in collaborative testing than in traditional testing.<br>Themes: (1) studying more/studying differently, (2)/ cognitive collectivism (3), "it stuck in my head better" (4), confidence, and (5) practicing how to share knowledge and negotiate.	moderate
Moineau et al., 2011,[42]<br>Canada<br><br>*Medical Education*	Compare scores and experiences of formative assessment from faculty and senior students during OSCE-examinations	Quantitative<br>Cross sectional<br>Pre- and post-questionnaire	n=66 medical students in year 2<br>n=27 year 4 student examiners<br>n=27 teaching doctors	Students (year 4) assessing students (year 2) with checklists in OSCE-examinations equally assessed compared to faculty members. A positive learning experience expressed from both students and faculty.	moderate
Nofziger et al., 2010,[37]	Investigate the impact of peer assessment on future	Qualitative	n=70 medical students in year 2	67% found peer assessment helpful, reassuring, or confirming something they	moderate

For peer review only - http://bmjopen.bmj.com/site/about/guidelines.xhtml

USA<br><br>*Academic Medicine*	professional development and students' experiences	Questionnaire and narrative comments<br>Frequency count	n=48 in year 4	knew; 65% reported important transformations in awareness, attitudes, or behaviors because of peer assessment. Change was more likely when feedback was specific and described an area for improvement.	
Rees, Sheard, & McPherson, 2002,[46]<br>United Kingdom<br><br>*Medical Education*	Explore students' perceptions of communication skill assessment	Qualitative<br>Focus groups	n=7 medical students in year 1<br>n= 7 in year 2<br>n=10 in year 3<br>n= 5 in year 4<br>n=3 in year 5	Year 4 and 5 more positive than younger students. Opportunities to compare communication skills with peers from same level. Learning experience being the assessor. No constructive criticism from peers. Difficult to be objective and to give feedback.	high
Satterthwaite & Grey, 2008,[43]<br>United Kingdom<br><br>*European Journal of Dental Education*	Investigate if any differences existed between marks given by a peer group and those given by experienced assessors	Quantitative<br>Cross sectional	n=65 dental students	No significant difference in grades between experienced examiners and peer group.	moderate
Spandorfer et al., 2014,[47]<br>USA<br><br>*Anatomical Science Education*	Determine whether peer assessment improves students work habits and interpersonal attributes and whether it is accepted by students, focusing on low performing students	Multi-methods<br>Paired sample t-test<br>Pearson correlation coefficients<br>Survey- content analysis	n=267 medical students in year 1; follow-up in year 2	Significant improvement after on-line peer feedback between test 1 and 2.<br>Themes: (1) Initiative, (2) Communication, (3) Respect, (4) Preparation, and (5) Focus. Students prefer anonymous feedback from peers.	moderate
Tai et al., 2016,[40]<br>Australia<br><br>*Advances in Health Science Education*	Investigate students' experience of peer-assisted learning.	Mixed methods<br>Ethnographic<br>Survey, observations, and interviews<br>Thematic analysis	n=10 medical students in year 1 (observed)<br><br>n=191 students in year 3 (survey)	Observing and giving feedback to peers contributed to learning, but students value feedback from teachers for validation. Students want to preserve social relationships with peers; therefore, feedback is not so constructive. Peers provide a supportive learning environment.	high
Tricio, Woolford, & Escudier, 2016,[45]<br>United Kingdom<br><br>*European Journal of Dental Education*	Analyze written feedback provided as a part of a formative and structured peer assessment protocol.	Multi-methods<br>Descriptive statistic<br>Thematic analysis	n=40 dental students in year 2 in pre-clinical skills laboratory<br><br>n=68 dental students in year 5 in clinic	Year 2 focuses on practical and clinical knowledge; in contrast, year 5 focuses comments on communication, management, and leadership. Year 2 gives more positive comments on peer performance than year 5.	moderate

For peer review only - http://bmjopen.bmj.com/site/about/guidelines.xhtml

Vaughn et al., 2016,[44] USA<br><br>*The American Journal of Surgery*	Evaluate the use, quality, and quantity of peer video feedback and compare peers and faculty feedback.	Quantitative<br>Cross sectional<br>Paired t-test, Mann-Whitney statistic<br>Survey	n=24 medical students***	Significant change in performance across 3 periods in both groups. Peer feedback group performed better at final assessment than faculty feedback group (not significant). Peers gave higher scores than faculty. No significant differences when using a checklist.	moderate

*High equals majority of items in the critical appraisal tools.

**24 students included in the intervention, and 19 attended the focus group session.

***12 students received faculty feedback, and 12 students received peer feedback.

For peer review only - http://bmjopen.bmj.com/site/about/guidelines.xhtml

Table 3. Overview and summery of the context, rationale, and interventions of formative peer assessment presented in the included studies.

Contexts	Rationales	Interventions
Intra-professional students (17)*<br>Combination of medical and nursing students (1)<br><br>Conducted in the following:<br>Clinical skill labs (11)<br>Theoretical courses (7)<br>Combination of theoretical and clinical placement course (1)<br><br>Within an educational model as problem-based learning, peer learning, or peer assisted learning (7)	Giving and receiving feeback supports student learning:<br>Promotes learning (8)<br>Enhances critical thinking (1)<br>Promotes understanding of the assessment process (1)<br>Develops critical- and interpersonal skills (1)<br>Helps identify knowledge gaps (1)<br>Supports low-performance students (1)<br><br>It prepares students for knowledge-related professionalism in the healthcare profession by helping them identify the following:<br>Professional- and un-professional behavior (6)<br>Clinical competence (2)<br>Technical skills (2)<br>Communication skills (2)<br>Collaborative behavior (2)<br>Evaluative judgement (1)<br><br>It enhances teachers' teaching (1)<br><br>It provides cost benefits:<br>Students as assessors instead of teachers (2)<br>Students as creators of the learning activities instead of teachers (1)	Introduction (in workshops):<br>Preparations in giving and receiving feedback (3)<br>Introduction of guidelines or checklists to guide the peer assessor (3)<br>Introduction of the learning activity (2)<br>Preparation in communication (1)<br><br>Learning activities focusing feedback on professionalism:<br>Clinical skills (3)<br>Collaborative behavior (2)<br>Clinical reasoning (2)<br>Theoretical knowledge (2)<br>Communication skills (2)<br>Management skills (1)<br><br>Feedback types:<br>Face-to-face (7)<br>Anonymous (5)<br>Written (3) or through observations (3)<br>Interactive on-line assessment (3)<br>Grading of the given feedback (1)<br><br>Random peers (8)<br>Ability to choose peer (1)<br>In small groups < 6 (6)<br>In large groups > 6 (3)

* = appears in how many of the included 18 studies

For peer review only

For peer review only - http://bmjopen.bmj.com/site/about/guidelines.xhtml

For peer review only

For peer review only - http://bmjopen.bmj.com/site/about/guidelines.xhtml

Figure 1: PRISMA Flow Diagram

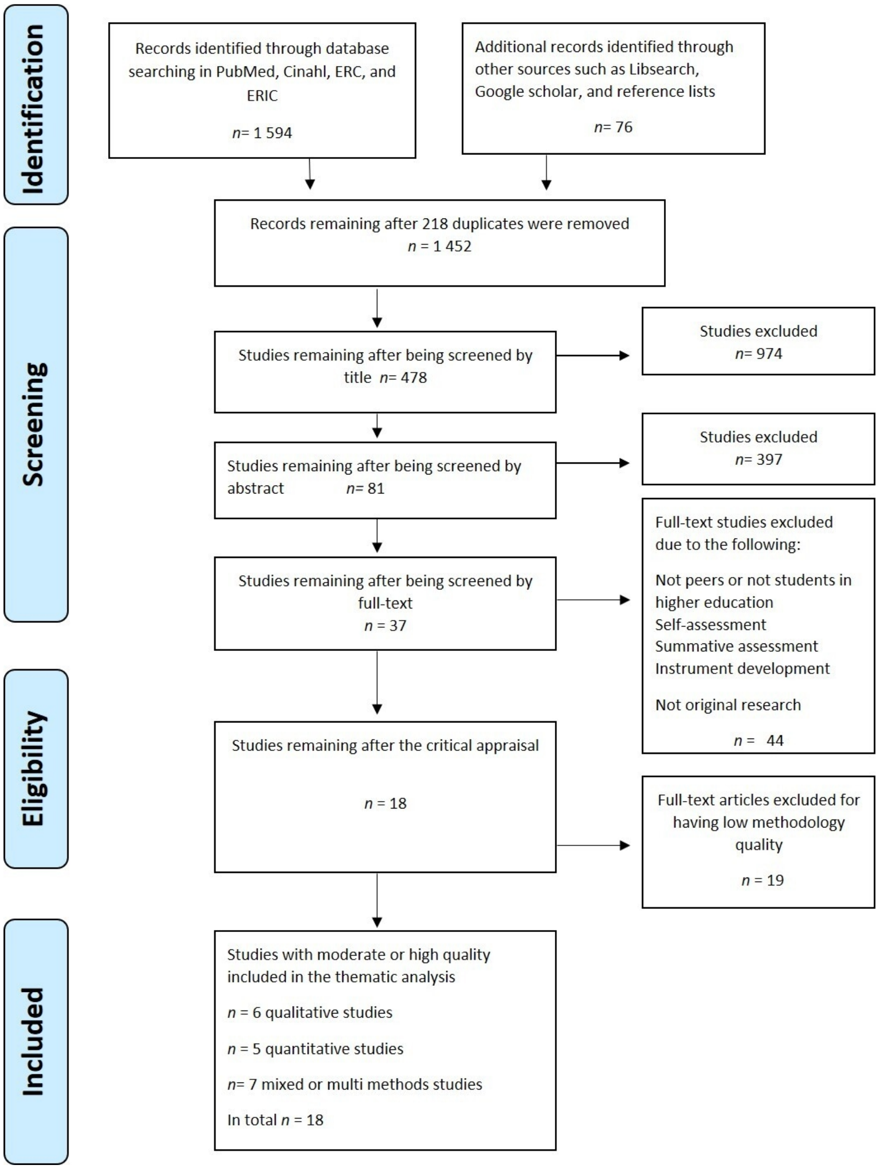

100x122mm (300 x 300 DPI)

Appendix 1. Presentation of the search strategy and results from the Pub Med database until 28th of May, 2019.

	Search block 1: Healthcare education
#	MESH-terms
1	"Students, Medical"[Mesh] OR
2	"Students, Nursing"[Mesh] OR
3	"Students, Dental"[Mesh] OR
4	"Students, Health Occupations"[Mesh] OR
5	"Education, Medical"[Mesh] OR
6	"Education, Nursing"[Mesh] OR
7	"Education, Dental" [Mesh] OR
8	"Midwifery/education"[Mesh] OR
9	"Allied Health Personnel/education"[Mesh]
10	S1 OR S2 OR S3 OR S4 OR S5 OR S6 OR S7 OR S8 OR S9
#	Free text terms
11	"medical student*" OR
12	"nursing student*" OR
13	"midwifery student* OR
14	"dental student*" OR
15	"physical therapy student*" OR
16	"occupational therapy student*" OR
17	"allied health student*" OR
18	"health occupations student*" OR
19	"health care stud*" OR
20	"Health care education" OR
21	"health science education" OR
22	"Medical education" OR
23	" Nursing education" OR
24	"Dental education" OR
25	"allied health education" OR
26	"Health occupation* education*" OR
27	"midwifery education"
28	S11 OR S12 OR S13 OR S14 ORS15 OR S16 OR S17 OR S18 OR S19 OR S20 OR S21 OR S22 OR S23 OR S24 OR S25 OR S26 OR S27
29	S10 OR S28

	Search block 2: Peer assessment
#	MESH
31	"Educational Measurement"[Mesh] OR
32	"Peer Group"[Mesh] OR
33	"Peer Review"[Mesh]
34	S31 OR S32 OR S33
#	Free text terms
35	"peer assessment" OR
36	"peer evaluation" OR
37	"peer observation" OR
38	"peer feedback" OR
39	"peer review" OR
40	"peer assess*" OR
41	"*peer assess" OR
42	"peer examiner" OR
43	"peer grad*" OR
44	"peer group" OR
45	"Student performance appraisal" OR
46	"educational measurement"
47	S35 OR S36 OR S37 OR S38 OR S39 OR S40 OR S41 OR S42 OR S43 OR S44 OR S45 OR S46
48	S34 OR S47

For peer review only

	Search block 3: Formative assessment
#	MESH
49	"Formative Feedback"[Mesh]
#	Free text terms
50	"Formative evaluation" OR
51	"Formative feedback" OR
52	"Formative assessment" OR
53	"Formative* assess*" OR
54	"Formativ* evaluation" OR
55	Formativ* OR
56	"formative evaluation research"
57	S37 OR S38 OR S39 OR S40 OR S41 OR S42 OR S43
58	S49 OR S57

#	Combination of search blocks 1, 2, and 3
59	S29 AND S48 AND S58

For peer review only

Appendix 2. Charting form

CHARTING FORM

ARTICLE NO:

TITLE:

AUTHOR/S:

YEAR OF PUBLICATION:

COUNTRY:

RESEARCH DESIGN: QUANTITATIVE ☐ QUALITATIVE ☐ OTHER: ☐ ____________

METHOD:

AIM:

NUMBER OF PARTICIPANTS (n=):

SETTING:

MEDICAL EDUCATION ☐

NURSING EDUCATION ☐

MIDWIFERY EDUCATION ☐

PHYSIOTHERAPY EDUCATION ☐

OCCUPATIONAL THERAPY ☐

DENTAL EDUCATION ☐

OTHER ☐ ____________

For peer review only

DOES THE STUDY PRESENT?

HOW PEER ASSESSEMENT INTERVENTIONS ARE DELIVERED	YES	NO	
PEER ASSESSMENT	YES	NO	
FORMATIVE ASSESSMENT	YES	NO	
STUDENTS EXPERIENCE	YES	NO	
TEACHERS EXPERIENCE	YES	NO	OTHER:
OUTCOME/S OF PEER ASSESSMENT INTERVENTION	YES	NO	
RATIONALE/S FOR PEER ASSESSMENT INTERVENTION	YES	NO	

MAIN FINDINGS:

INCLUDED: YES NO

REASON/S FOR EXCLUSION:

For peer review only

(Only to be answered if the study is to be included)

Grading according to the Critical Appraisal Skills Program (CASP)

QUALITATIVE STUDIES AND SYSTEMATIC REVIEWS

LOW ☐ MODERATE ☐ HIGH ☐

RCT AND CASE CONTROL STUDIES

LOW ☐ MODERATE ☐ HIGH ☐

COHORT STUDIES

LOW ☐ MODERATE ☐ HIGH ☐

Grading according to The Mixed Method Appraisal Tool

MIXED METHOD STUDIES

LOW ☐ MODERATE ☐ HIGH ☐

Grading according to the Joanna Briggs Institute Critical Appraisal Tool

QUASI-EXPERIMENTAL STUDIES

LOW ☐ MODERATE ☐ HIGH ☐

ANALYTICAL CROSS SECTIONAL STUDIES

LOW ☐ MODERATE ☐ HIGH ☐

CASE REPORTS

LOW ☐ MODERATE ☐ HIGH ☐

Reviewed by:

(Signature and date)

For peer review only

Preferred Reporting Items for Systematic reviews and Meta-Analyses extension for Scoping Reviews (PRISMA-ScR) Checklist

SECTION	ITEM	PRISMA-ScR CHECKLIST ITEM	REPORTED ON PAGE #
**TITLE**			
Title	1	Identify the report as a scoping review.	1
**ABSTRACT**			
Structured summary	2	Provide a structured summary that includes (as applicable): background, objectives, eligibility criteria, sources of evidence, charting methods, results, and conclusions that relate to the review questions and objectives.	2
**INTRODUCTION**			
Rationale	3	Describe the rationale for the review in the context of what is already known. Explain why the review questions/objectives lend themselves to a scoping review approach.	3, 4
Objectives	4	Provide an explicit statement of the questions and objectives being addressed with reference to their key elements (e.g., population or participants, concepts, and context) or other relevant key elements used to conceptualize the review questions and/or objectives.	5
**METHODS**			
Protocol and registration	5	Indicate whether a review protocol exists; state if and where it can be accessed (e.g., a Web address); and if available, provide registration information, including the registration number.	4,13
Eligibility criteria	6	Specify characteristics of the sources of evidence used as eligibility criteria (e.g., years considered, language, and publication status), and provide a rationale.	5, Table 1
Information sources*	7	Describe all information sources in the search (e.g., databases with dates of coverage and contact with authors to identify additional sources), as well as the date the most recent search was executed.	6, Appendix 1
Search	8	Present the full electronic search strategy for at least 1 database, including any limits used, such that it could be repeated.	Appendix 1
Selection of sources of evidence†	9	State the process for selecting sources of evidence (i.e., screening and eligibility) included in the scoping review.	6,7
Data charting process‡	10	Describe the methods of charting data from the included sources of evidence (e.g., calibrated forms or forms that have been tested by the team before their use, and whether data charting was done independently or in duplicate) and any processes for obtaining and confirming data from investigators.	6,7, Appendix 2
Data items	11	List and define all variables for which data were sought and any assumptions and simplifications made.	5, Appendix 1
Critical appraisal of individual sources of evidence§	12	If done, provide a rationale for conducting a critical appraisal of included sources of evidence; describe the methods used and how this information was used in any data synthesis (if appropriate).	6, 7

For peer review only - http://bmjopen.bmj.com/site/about/guidelines.xhtml

SECTION	ITEM	PRISMA-ScR CHECKLIST ITEM	REPORTED ON PAGE #
Synthesis of results	13	Describe the methods of handling and summarizing the data that were charted.	6,7
**RESULTS**			
Selection of sources of evidence	14	Give numbers of sources of evidence screened, assessed for eligibility, and included in the review, with reasons for exclusions at each stage, ideally using a flow diagram.	5,6, Figure 1
Characteristics of sources of evidence	15	For each source of evidence, present characteristics for which data were charted and provide the citations.	7, Table 2
Critical appraisal within sources of evidence	16	If done, present data on critical appraisal of included sources of evidence (see item 12).	Table 2
Results of individual sources of evidence	17	For each included source of evidence, present the relevant data that were charted that relate to the review questions and objectives.	7,8,9,10, Table 2
Synthesis of results	18	Summarize and/or present the charting results as they relate to the review questions and objectives.	8,9,10, Table 3
**DISCUSSION**			
Summary of evidence	19	Summarize the main results (including an overview of concepts, themes, and types of evidence available), link to the review questions and objectives, and consider the relevance to key groups.	10, 11
Limitations	20	Discuss the limitations of the scoping review process.	12
Conclusions	21	Provide a general interpretation of the results with respect to the review questions and objectives, as well as potential implications and/or next steps.	11, 12
**FUNDING**			
Funding	22	Describe sources of funding for the included sources of evidence, as well as sources of funding for the scoping review. Describe the role of the funders of the scoping review.	12

JBI = Joanna Briggs Institute; PRISMA-ScR = Preferred Reporting Items for Systematic reviews and Meta-Analyses extension for Scoping Reviews.
* Where *sources of evidence* (see second footnote) are compiled from, such as bibliographic databases, social media platforms, and Web sites.
† A more inclusive/heterogeneous term used to account for the different types of evidence or data sources (e.g., quantitative and/or qualitative research, expert opinion, and policy documents) that may be eligible in a scoping review as opposed to only studies. This is not to be confused with *information sources* (see first footnote).
‡ The frameworks by Arksey and O'Malley (6) and Levac and colleagues (7) and the JBI guidance (4, 5) refer to the process of data extraction in a scoping review as data charting.
§ The process of systematically examining research evidence to assess its validity, results, and relevance before using it to inform a decision. This term is used for items 12 and 19 instead of "risk of bias" (which is more applicable to systematic reviews of interventions) to include and acknowledge the various sources of evidence that may be used in a scoping review (e.g., quantitative and/or qualitative research, expert opinion, and policy document).

From: Tricco AC, Lillie E, Zarin W, O'Brien KK, Colquhoun H, Levac D, et al. PRISMA Extension for Scoping Reviews (PRISMA-ScR): Checklist and Explanation. Ann Intern Med. ;169:467–473. doi: 10.7326/M18-0850

For peer review only - http://bmjopen.bmj.com/site/about/guidelines.xhtml

BMJ Open

Formative Peer Assessment in Higher Healthcare Education Programs – a Scoping Review

Journal:	*BMJ Open*
Manuscript ID	bmjopen-2020-045345.R2
Article Type:	Original research
Date Submitted by the Author:	29-Dec-2020
Complete List of Authors:	Stenberg, Marie; Malmo Universitet, Care Science<br>Mangrio, Elisabeth; Malmo Universitet, Care Science<br>Bengtsson, Mariette; Malmo Universitet, Care Science<br>Carlson, Elisabeth; Malmo Universitet,
**Primary Subject Heading**:	Medical education and training
Secondary Subject Heading:	Medical education and training
Keywords:	MEDICAL EDUCATION & TRAINING, EDUCATION & TRAINING (see Medical Education & Training), Quality in health care < HEALTH SERVICES ADMINISTRATION & MANAGEMENT

SCHOLARONE™
Manuscripts

I, the Submitting Author has the right to grant and does grant on behalf of all authors of the Work (as defined in the below author licence), an exclusive licence and/or a non-exclusive licence for contributions from authors who are: i) UK Crown employees; ii) where BMJ has agreed a CC-BY licence shall apply, and/or iii) in accordance with the terms applicable for US Federal Government officers or employees acting as part of their official duties; on a worldwide, perpetual, irrevocable, royalty-free basis to BMJ Publishing Group Ltd ("BMJ") its licensees and where the relevant Journal is co-owned by BMJ to the co-owners of the Journal, to publish the Work in this journal and any other BMJ products and to exploit all rights, as set out in our licence.

The Submitting Author accepts and understands that any supply made under these terms is made by BMJ to the Submitting Author unless you are acting as an employee on behalf of your employer or a postgraduate student of an affiliated institution which is paying any applicable article publishing charge ("APC") for Open Access articles. Where the Submitting Author wishes to make the Work available on an Open Access basis (and intends to pay the relevant APC), the terms of reuse of such Open Access shall be governed by a Creative Commons licence – details of these licences and which Creative Commons licence will apply to this Work are set out in our licence referred to above.

Other than as permitted in any relevant BMJ Author's Self Archiving Policies, I confirm this Work has not been accepted for publication elsewhere, is not being considered for publication elsewhere and does not duplicate material already published. I confirm all authors consent to publication of this Work and authorise the granting of this licence.

For peer review only

Formative Peer Assessment in Higher Healthcare Education Programs – a Scoping Review

Marie Stenberg[1] RN; MNSc marie.stenberg@mau.se

Elisabeth Mangrio[1] RN, PhD, Associate Professor elisabeth.mangrio@mau.se

Mariette Bengtsson[1] RN, PhD, Associate Professor mariette.bengtsson@mau.se

Elisabeth Carlson[1] RN, PhD, Professor elisabeth.carlson@mau.se

[1]Department of Care Science, Faculty of Health and Society, Malmö University, SE 205 06 Sweden

Corresponding author: Elisabeth Carlson, Faculty of Health and Society, Jan Waldenströmsg 25, Malmö University, SE 214 28 Malmö, Sweden

Tel: +46 40 665 79 44

E-Mail: marie.stenberg@mau.se

Word count: 3746

For peer review only

ABSTRACT

Objectives: Formative peer assessment focus on learning and development of the student learning process. This implies that students are taking responsibility for assessing the work of their peers by giving and receiving feedback to each other. The aim was to compile research about formative peer assessment presented in higher healthcare education, focusing on the rationale, the interventions, the experiences of students and teachers and the outcomes of formative assessment interventions.

Design: A scoping review.

Data sources: Searches were conducted until May 2019 in PubMed, CINAHL, ERIC, and ERC. Grey literature was searched in Lib-search, Google Scholar and Science Direct.

Eligibility criteria: Studies addressing formative peer assessment in higher education, focusing on medicine, nursing, midwifery, dentistry, physical or occupational therapy, and radiology published in peer reviewed articles or in grey literature.

Data extractions and synthesis: Out of 1452 studies, 37 met the inclusion criteria and were critically appraised using relevant CASP, JBI and MMAT tools. The pertinent data was analyzed using thematic analysis.

Result: The critical appraisal resulted in 18 included studies with high and moderate quality. The rationale for using formative peer assessment relates to giving and receiving constructive feedback as a means to promote learning. The experience and outcome of formative peer assessment interventions from the perspective of students and teachers are presented within three themes; 1/organization and structure of the formative peer assessment activities, 2/ personal attributes and consequences for oneself and relationships and 3/ experience and outcome of feedback and learning.

Conclusion: Healthcare education must consider preparing and introducing students to collaborative learning, and thus develop well-designed learning activities aligned with the learning outcomes. Since peer collaboration seems to affect students' and teachers' experiences of formative peer assessment, empirical investigations exploring collaboration between students are of utmost importance.

Keywords: Feedback, formative assessment, healthcare education, peer assessment, students, teachers

For peer review only

STRENGTHS AND LIMITATIONS OF THE STUDY

- The current scoping review is previously presented in a published study protocol.
- Four databases were systematically searched to identify research on formative peer assessment.
- Critical appraisal tools were used to assess the quality of studies with quantitative, qualitative, and mixed methods designs.
- Articles appraised as high or moderate quality were included.
- Since only English studies were included, studies may have been missed that would otherwise have met the inclusion criteria.

BACKGROUND

Peer assessment is an educational approach where feedback, communication, reflection, and collaboration between peers are key characteristics. In a peer assessment activity, students take responsibility for assessing the work of their peers by giving (and receiving) feedback on a specific subject.[1] It allows students to consider the learning outcomes for peers of similar status and to reflect upon their own learning mirrored in a peer.[2] Peer assessment has shown to support students' development of judgement skills, critiquing abilities, and self-awareness as well as their understanding of the assessment criteria used in a course.[1] In higher education, peer assessment has been a way to move from an individualistic and teacher-led approach to a more collaborative, student-centred approach to assessment[1] aligned with social constructivism principles.[3] In this social context of interaction and collaboration, students can expand their knowledge, identify their strengths and weaknesses, and develop personal and professional skills[4] by evaluating the professional competence of a peer.[5] Peer assessment can be used in academic and professional settings as a strategy to enhance students' engagement in their own learning.[6 7 8] The collaborative aspect of peer assessment relates to professional teamwork, as well as to broader goals of lifelong learning. As argued by Boud et al.,[1] peer assessment addresses course-specific goals not readily developed otherwise. For healthcare professions, it enhances the ability to work in a team in a supportive and respectful atmosphere,[9] which is highly relevant for patient outcome and the reduction of errors

For peer review only - http://bmjopen.bmj.com/site/about/guidelines.xhtml

compromising patient safety.[10] However, recent research has shown that peer collaboration is challenging[11] and that healthcare professionals are not prepared to deliver and receive feedback effectively.[12] This emphasizes the importance for healthcare educators to support students with activities fostering these competences. Feedback is highly associated with enhancing student learning[13] and modifying learning during the learning process[14] as a means for students to close the gap between their present state of learning and their desired goal(s). Peer feedback can be written or oral and conducted as peer observations in small or large groups.[8] Further, it is driven by set assessment criteria,[1] which can be either summative or formative, formal or informal. Summative assessment evaluates students' success or failure after the learning process,[15] whereas formative assessment aims for improvement during the learning process.[16,4] According to Black and Wiliam,[15] formative peer assessment activities involve feedback to modify the teaching and learning of the students. The intention of feedback is to help students help each other when planning their learning.[4 17] An informal formative peer assessment activity involves a continuous process throughout a course or education, whereas a formal one is designated to a single point in a course momentum. Earlier research on peer assessment in healthcare education has provided an overview of specific areas within the peer assessment process. For example, Speyer, Pilz, and Van Der Kruis presented psychometric characteristics of peer assessment instruments and questionnaires in medical education,[18] concluding that quite a few instruments exist; however, these intruments mainly focus on professional behavior, and they lack sufficient psychometric data. Tornwall[12] focused on how nursing students were prepared by academics to participate in peer assessment activities and highlighted the importance of creating a supporting learning environment. Lerchenfeldt, Mi and Eng[19] concluded that peer assessment supports medical students in developing professional behavior and that peer feedback is a way to assess professionalism. Khan, Payne, and Chahine[20] reviewed the role of peer assessment in objective structured clinical examinations (OSCE), showing that peer assessment promotes learning but that students need training in how to provide feedback. In short, the existing literature contributes valuable knowledge about formative peer assessment in healthcare education targeting specific areas. However, there seems to be a lack of compiled research considering formative peer assessment in its entirety, including the context, rationale, experience, and outcome of the formative peer assessment process. Therefore, this scoping review attempts to present an overview of formative peer assessment in healthcare education rather than specific areas within that process.

For peer review only - http://bmjopen.bmj.com/site/about/guidelines.xhtml

METHOD

This scoping review was conducted using the York methodology by Arksey and O'Malley [21] and the recommendations presented by Levac, Colquhuan, and O'Brien.[22] We constructed a scoping protocol, utilizing a PRISMA-P protocol, to present the planned methodology for the scoping review.[23]

Aim and research questions

We aimed to compile research about formative peer assessment presented in higher healthcare education. The research questions were as follows: What are the rationales for using formative peer assessment in healthcare education? How are formative peer assessment interventions delivered in healthcare education and in what context? What experiences of formative peer assessment do students and teachers in healthcare education have? What are the outcomes of formative peer assessment interventions? We used the "Population Concept and Context" (PCC) elements recommended for scoping reviews to establish effective search criteria (Table 1).[24]

INSERT TABLE 1 HERE

Relevant studies identified

The literature search was conducted in the databases PubMed, Cumulative Index to Nursing and Allied Health Literature (CINAHL), Education Research Complete (ERIC), and Education Research Centre (ERC). Search tools such as Medical Subject Headings, Headings, Thesaurus and Boolean operators (AND/OR) helped expand and narrow the search. Initially, the search terms were broad (e.g., peer assessment or higher education) in order to capture the range of published literature. However, the extensiveness of the material made it necessary to narrow the search terms and organize them in three major blocks. The following inclusion criteria were applied in the search: (a) articles addressing formative peer assessment in higher education; (b) students and teachers in medicine, nursing, midwifery, dentistry, physical or occupational therapy, and radiology; and (c) peer reviewed articles, grey literature (books, discussion papers, posters et cetera). Studies of summative peer assessment, instrument development, and systematic reviews were excluded. We incorporated several similar terms related to peer assessment in the search to ensure that no studies were missed (Appendix 1). Furthermore, we consulted a well-versed librarian with experience of systematic search[25] to assist us in systematically identifying relevant databases and search terms for each database,

For peer review only

control the relevance of the constructed search blocks, and manage the data in a reference management system. No limitation was set for year, all studies indexed in the four databases were included until the last search May 28th, 2019.

Study selection

The process of the study selection and the reasons for exclusion are presented in a flow diagram[26] (Figure 1). First, the first author (MS) screened all 1,452 titles. Second, MS read all the abstracts, gave those responding to the research questions a unique code, and organized them in a reference management system. The reason for inclusion and exclusion at title and abstract level was charted by the first author and critically discussed within the team (MS, EM, MB, EC). An additional hand search of reference lists was conducted. To cover a subject in full, a scoping review should include search in grey literature.[21 22] Therefore, the grey literature was scoped to find unpublished results by searching Google Scholar, LibSearch, and Science Direct. The grey literature mostly contained research posters, conference abstracts, discussion papers, and books, but a hand search revealed original research articles that were added for further screening and appraisal. Finally, the first author (MS) arrived at 81 studies, read them in full-text, and discussed them with the other three authors (EM, MB, EC).

INSERT FIGURE 1 HERE

Charting the data

We constructed a charting form to facilitate the screening of the full-text studies (Appendix 2). Out of the 81 studies, 37 met the inclusion criteria and were appraised for quality using Critical Appraisal Skills Programme (CASP).[27] The reason for conducting a crtitical appraisal of the studies was to enhance the use of the findings for policy-making and practice in higher healthcare education.[28] To investigate the interpretation of the quality instruments, three members of the research team (MS, EM, EC) conducted an initial test assessment of two randomly selected studies and graded them with high, moderate, or low quality. Additional screening tools were used for studies with a mixed methods design[29] and cross-sectional studies[30] not available in CASP. When a discrepancy arose, a fourth researcher (MB) assessed the articles independently without prior knowledge of what the others have concluded. This was followed by a discussion among all four researchers to secure internal agreement on how to further interpret the checklist items and the quality assessments. Consequently, to ensure high quality, the studies had to have a "yes" answer for a majority of the questions. If "no" dominated, the study was excluded. Since earlier reports[31] have raised and discussed the

For peer review only - http://bmjopen.bmj.com/site/about/guidelines.xhtml

For peer review only

importance of ethical issues in systematic reviews, all screening protocols in this review included ethical considerations, as an individual criterion. The first author critically appraised all 37 articles, and 15 articles were divided between the team members (EM, MB, EC) and independently appraised. Nevertheless, during the screening process all 37 articles were critically discussed using the Rayyan system for systematic reviews[32] before final descision for inclusion. By this procedure, all authors agreed on not only which articles to include, but also the reason for exclusion. The critical appraisal resulted in 18 studies with high and moderate quality (Table 2).

INSERT TABLE 2 HERE

Collating, summarizing, and reporting results

The analysis process followed the five phases of thematic analysis described by Braun and Clarke,[33] with support of a practical guide provided by Maquire & Delahunt.[34] The first phase included familiarizing with the data. Therefore, prior to the coding process, we read all the articles to grasp a first impression of the results presented within the included studies.We then conducted a theoretical thematic analysis, meaning that the results were deductively coded,[33] guided by the research questions. We read the results a second time before starting the initial coding. The codes consisted of short descriptions close to the original text. The codes were then combined into themes and sub-themes. The themes were identified with a semantic approach, meaning that they were explicit: we did not look for anything beyond what was written.[33] Finally, we constructed a thematic map to present an overview of the results and how the themes related to each other. The results from the studies are presented narratively.

Consultation

Consultation is an optional stage in scoping reviews.[21] However, since it adds methodologic rigor,[22] we presented and discussed the preliminary results and the thematic map with nine academic teachers who are experts within the field of healthcare education and pedagogy. The purpose of the consultation was to enhance the validity of the results of the scoping review and to facilitate appropriate dissemination of outputs.[33] The expert group responded to four questions: Do the themes make sense? Is too much data included in one single theme? Are the themes distinct or do they overlap? Are there themes within themes?[34] The consultation resulted in a revision of a few themes and the way they related to each other.

For peer review only - http://bmjopen.bmj.com/site/about/guidelines.xhtml

RESULTS

The 18 included studies were published between 2002 and 2017 in the United States (6), the United Kingdom (6), Australia (3), Canada (2), and the United Arab Emirate (1) (Table 3). The studies were conducted in medical (12), dental (2), nursing (2), occupational therapy (1), and radiography (1) educations. Six studies were presented in the framework of an existing collaborative educational model.[35-37 38 39 40] Our review revealed that the most frequent setting for formative peer assessment activities is within clinical skill-training courses,[35 39-47] involving intra-professional peers. The common rationale for using formative peer assessment is to support students, usually explained by the inherent learning of the feedback process,[35 39 40 43-45 47-51] and to prepare students for professional behavior and provide them with the skills required in the health care professions.[36 37 38 46 47 48 49 52] Table 3 presents the results of the analysis related to the research questions of context, rationale, and interventions of formative peer assessment.

INSERT TABLE 3 HERE

The results related to the research questions about the experience of students and teachers and the outcome of formative peer assessment interventions fall within three themes: (1) the organization and structure of peer assessment activities, (2) personal attributes and consequences for oneself and one's peer relationships, and (3) the experience and outcome of feedback and learning.

The organization and structure of formative peer assessment activities

In the reviewed studies, students express that the responsibility of faculty is a key component in formative peer assessment, meaning that faculty must clearly state the aim of the peer assessment activity. Students highlight the need to be prepared and trained in how to give and receive constructive feedback.[36 47 50-52] The learning activities need to be well-designed and supported by guidelines on how to use them.[35 36 50 52] Otherwise, it could discourage students from participating in the peer activities.[52] Novice students find it difficult to be objective and to offer constructive criticism in a group.[36 46] This emphasizes the importance of responsibility from faculty, especially when students are to give feedback on professional behavior.[52] Some students prefer direct communication with peers when feedback is negative, whereas others think it is the responsibility of faculty.[52] There is some ambiguity regarding whether feedback should be given

For peer review only - http://bmjopen.bmj.com/site/about/guidelines.xhtml

anonymously or not,[47 52] whether it should bear consequences from faculty or not,[52] whether it should be informal or formal, and whether the peer should be at the same academic level or at a more experienced higher-level.[50 52] Moreover, some students express how they favor small groups;[41 49] as students in small groups are more active than those in large groups.[41] Students and teachers agree that peer assessment should be strictly formative rather than summative.[42 46 52] Teachers see themselves as key facilitators and express that students value feedback from teachers rather than from peers (in terms of credibility).[51] Students express similar sentiments even if they appreciate the peer feedback.[40 42 44 46] However, teachers confirm the need for training and preparing students early in the education, as well as the need for their own professional development to guide students effectively.[51]

Personal attributes and the impact and consequences for oneself and one's peer relationships

Students generally focus on how peer assessment activities may affect their personal relationships in a negative way.[35 37 42 50 52] They express worry over consequences for themselves and their social relationships[37 40 52] as well as feeling anxious that negative feedback given to a peer may affect the grading from faculty.[52] Moreover, students emphasize the importance of enthusiasm and engagement in listening to peers' opinions during their collaboration.[36 47] They mention positive personal attributes and behaviors such as being organized, polite, and helpful as supportive for peer collaboration.[36 47] Further, they mention the importance of both a positive and close relationship between students and faculty[52] and a positive culture in the learning environment.[40] While students highlight the impact on and consequences for personal relationships, teachers speak of the importance of respect in formative peer assessment,[36] including respect for each other, the learning activity, and the collaboration and interaction.[36] Further, teachers emphasize the importance of students being self-aware, being well prepared, and taking own responsibility for the peer assessment activity.[36]

The experience and outcome of feedback and learning

According to the students in the reviewed studies, formative peer assessment contributes to developing the skills needed in practice and in their future profession.[35 36 40 41 48 52] They appreciate the opportunity to give and receive feedback from a peer,[35 36 40 42 47 48 50] and they agree that the feedback they received made them change how they worked[42 48] or

For peer review only - http://bmjopen.bmj.com/site/about/guidelines.xhtml

how they taught their peers.[47 48] They consider activities such as observation of others' performance as beneficial for learning because they make them reflect on their own performance[35 36 40 41 46 49 50] and help them identify knowledge gaps.[35 40 49] Students with prior experience of peer learning are more likely to provide specific guiding feedback than those without such experiences.[39] Moreover, two studies showed significantly improved test results for students who took part in a peer feedback activity compared to those who did not.[43 49] Further, students thought hey could be honest in their feedback and would learn better if the feedback was more in-depth.[35 46] Students at entry level tend to give more positive feedback than senior students; they also focus on practical and clinical knowledge, whereas more senior students focus on communication, management, and leadership in their feedback comments.[45] A study exploring what students remember of received feedback points to memories of positive growth, negative self-image, and negative attitudes toward classmates. Received feedback sometimes confirmed personal traits the students already knew about.[37] In addition, negative feedback was more likely to result in a change in their work habits and interpersonal attributes.[37] Students expressed some anxiety regarding the usefulness of feedback from low-performing students[40 50] and non-motivated students, which contributes to ineffective interaction and learning.[36 47] Low performing students show lack of initiative, preparation, and respect but also improvement in their grades after the peer assessment experience.[47] Furthermore, feedback from peers can be a predictor of a student's unprofessional behavior; hence, it could be used as a tool for early remediation.[38] In an evaluation of faculty examiners' experience of students' feedback, the faculty express how they consider student feedback to be given in a professional and appropriate way and faculty examiners would have given similar feedback.[42] In an OSCE-examination where a checklist was used, the results showed statistical significance in assessment between faculty examiners and student examiners.[42]

DISCUSSION

We conclude that formative peer assessment is a process with two consecutive phases. The first phase concerns the understanding of the rationale and fundament of the peer assessment process for students and faculty members. The results indicate that the rationale is to support student learning and prepare them for healthcare professions. The formative peer assessment activities support students' reflection on their own knowledge and development when mirrored in a peer by alternating the roles of observer and observed. [53 54] It further contributes

For peer review only - http://bmjopen.bmj.com/site/about/guidelines.xhtml

to skills as communication, transfer of understandable knowledge and collaboration, all significant core competences when caring for patients and their relatives.[54] For faculty, organizing formative peer assessment, can be cost beneficial. This was recently emphasized in high volume classes expressing the reduction of costs with students giving feedback to a peer instead of teachers.[55] Nevertheless, students express the importance of clarifying the aim of the peer assessment activity and the responsibility of the faculty. We recommend faculty to clearly define the activity and explain how it supports student learning and professionalism, especially when students are to provide feedback to each other on sensitive matters, such as un-professional behavior. A collaborative activity between students requires trust, and the real intention must be made transparent. [4 56-58] Moreover, to enable student development in line with the learning outcomes, the learning activity needs to be well designed and understood by students.[59 60 61] However, Casey et al.[62] recommended further investigations of how to prepare students for the peer assessment activities.

The second phase concerns the organization and structure of the formative peer assessment activity, for example, how to give and receive feedback and the complexity of peer collaboration as it affects students' emotions concerning both themselves and their relationship with their peers. This coincides with earlier research emphasizing the social factors of peer assessment and the importance for teachers to consider them.[4] Nevertheless, surprisingly, few studies highlight the collaborative part of peer assessment.[4 11] One reason might be that formative peer assessment is often presented as a "stand alone" activity and not involved in a collaborative learning environment.[8 63] We agree with earlier research[64 65] arguing that peer assessment needs to be affiliated with practices of collaborative learning. Similar implications are presented by Tornwall,[12] who concluded the importance of integrating peer collaboration as a natural approach throughout education to support student development.

CONCLUSIONS AND IMPLICATIONS FOR FURTHER RESEARCH

Some have argued that research on peer assessment is deficient in referring to exactly what peer assessment aims to achieve.[66] We conclude that within healthcare education the aim of formative peer assessment is to prepare students for the collaborative aspects crucial within the healthcare professions. However, healthcare education must consider preparing and introducing students to collaborative learning; therefore, well-designed learning activities

For peer review only - http://bmjopen.bmj.com/site/about/guidelines.xhtml

aligned with the learning outcomes need to be developed. Based on this scoping review, formative peer assessment needs to be implemented in a collaborative learning environment throughout the education to be effective. However, since peer collaboration seems to affect students' and teachers' experience of formative peer assessment, empirical investigations exploring the collaboration between students are of utmost importance.

LIMITATIONS

Previous methodological concerns and discussions have been related to the systematic approach of handling grey literature.[67 68] We argue that the grey literature may contribute to a wider understanding of the research area. Nevertheless, when we conducted a critical appraisal of the included studies, the grey literature was excluded due to lack of methodological rigor. Therefore, we recommend considering this time-consuming phase of the methodology in scoping reviews. We further acknowledge that the last search was conducted in May 2019, studies may have been included if an additional search had been provided after this date and in other databases than the ones presented. Further, the current scoping review has not fully elucidated the perspective of teachers and faculty. Few of the included studies highlighted the teachers' perspective why further research is required.

Authors' contribution: MS led the design, search strategy, and conceptualization of this work and drafted the manuscript. EM, MB, and EC were involved in the conceptualization of the review design, inclusion and exclusion criteria, and critical appraisal and provided feedback on the methodology and the manuscript. All authors give their approval to the publishing of this scoping review manuscript.

Funding statement: This research received no specific grant from any funding agency in the public, commercial, or not-for-profit sectors.

Competing interests: None declared.

Patient and public involvement: As this is a scoping review no primary research has been conducted and thus not involve patients or the public.

Acknowledgement: Special thanks to the members in the expert group for their valuable contribution in the consultation.

Additional data: No data available. All data relevant to the study are included in the article or uploaded as supplementary information.

For peer review only - http://bmjopen.bmj.com/site/about/guidelines.xhtml

Figure 1. PRISMA Flowchart

REFERENCES

1. Boud D, Cohen R, Sampson J. Peer learning and assessment. *Assessment & evaluation in higher education* 1999;24(4):413-26.
2. Topping KJ, Ehly SW. Peer assisted learning: A framework for consultation. *Journal of Educational and Psychological Consultation* 2001;12(2):113-32.
3. Vygotskiĭ LS. Thought and language: MIT press 2012.
4. Topping KJ. Peer Assessment. 2009:20-27. doi: 10.1080/00405840802577569
5. Dannefer EF, Henson LC, Bierer SB, et al. Peer assessment of professional competence. *Medical Education* 2005;39(7):713-22. doi: 10.1111/j.1365-2929.2005.02193.x
6. Casey D, Burke E, Houghton C, et al. Use of peer assessment as a student engagement strategy in nurse education. *Nursing & health sciences* 2011;13(4):514-20.
7. Orsmond* P, Merry S, Callaghan A. Implementation of a formative assessment model incorporating peer and self-assessment. *Innovations in Education and Teaching International* 2004;41(3):273-90.
8. Ashenafi MM. Peer-assessment in higher education – twenty-first century practices, challenges and the way forward. *Assessment and Evaluation in Higher Education* 2017;42(2):226-51. doi: 10.1080/02602938.2015.1100711
9. Sims S, Hewitt G, Harris R. Evidence of collaboration, pooling of resources, learning and role blurring in interprofessional healthcare teams: a realist synthesis. *Journal of Interprofessional care* 2015;29(1):20-25.
10. Morley L, Cashell A. Collaboration in health care. *Journal of medical imaging and radiation sciences* 2017;48(2):207-16.
11. Stenberg M, Carlson E. Swedish student nurses' perception of peer learning as an educational model during clinical practice in a hospital setting—an evaluation study. *BMC nursing* 2015;14(1):48.
12. Tornwall J. Peer assessment practices in nurse education: An integrative review, 2018:266-75.
13. Nicol DJ, Macfarlane-Dick D. Formative assessment and self-regulated learning: A model and seven principles of good feedback practice. *Studies in higher education* 2006;31(2):199-218.
14. Hudson JN, Bristow DR. Formative assessment can be fun as well as educational. *Advances in physiology education* 2006;30(1):33-37. doi: 10.1152/advan.00040.2005
15. Black P, Wiliam D. Assessment and classroom learning. *Assessment in Education: principles, policy & practice* 1998;5(1):7-74.
16. Sadler DR. Beyond feedback: Developing student capability in complex appraisal. *Assessment & Evaluation in Higher Education* 2010;35(5):535-50.
17. Topping K. Peer assessment between students in colleges and universities. *Review of educational Research* 1998;68(3):249-76.
18. Speyer R, Pilz W, Van Der Kruis J, et al. Reliability and validity of student peer assessment in medical education: a systematic review. *Med Teach* 2011;33(11):e572-85. doi: 10.3109/0142159X.2011.610835 [published Online First: 2011/10/26]
19. Lerchenfeldt S, Mi M, Eng M. The utilization of peer feedback during collaborative learning in undergraduate medical education: a systematic review. *BMC medical education* 2019;19(1):321.
20. Khan R, Payne MWC, Chahine S. Peer assessment in the objective structured clinical examination: A scoping review. *Medical Teacher* 2017;39(7):745-56. doi: 10.1080/0142159X.2017.1309375
21. Arksey H, O'Malley L. Scoping studies: towards a methodological framework. *International Journal of Social Research Methodology* 2005;8(1):19-32. doi: 10.1080/1364557032000119616

For peer review only

For peer review only - http://bmjopen.bmj.com/site/about/guidelines.xhtml

22. Levac D, Colquhoun H, O'Brien KK. Scoping studies: advancing the methodology. *Implementation Science* 2010;5(1):69. doi: 10.1186/1748-5908-5-69
23. Stenberg M, Mangrio E, Bengtsson M, et al. Formative peer assessment in healthcare education programmes: protocol for a scoping review. *BMJ open* 2018;8(11):e025055-e55. doi: 10.1136/bmjopen-2018-025055
24. Peters M, Godfrey C, McInerney P, et al. Methodology for JBI scoping reviews. The Joanna Briggs Institute Reviewers manual 2015: The Joanna Briggs Institute 2015:3-24.
25. Morris M, Boruff JT, Gore GC. Scoping reviews: establishing the role of the librarian. *Journal of the Medical Library Association: JMLA* 2016;104(4):346.
26. Moher D, Liberati A, Tetzlaff J, et al. Preferred reporting items for systematic reviews and meta-analyses: the PRISMA statement. *Annals of internal medicine* 2009;151(4):264-69.
27. Programme CAS. Critical Appraisal Skills Programme. CASP checklist 2018 [Available from: https://casp-uk.net/casp-tools-checklists/ accessed 5 august 2018.
28. Daudt HM, van Mossel C, Scott SJ. Enhancing the scoping study methodology: a large, inter-professional team's experience with Arksey and O'Malley's framework. *BMC medical research methodology* 2013;13(1):48.
29. Hong Q, Pluye P, Fàbregues S, et al. Mixed Methods Appraisal Tool (MMAT) version 2018: User guide. *Department of Family Medicine, McGuill Univertiy* 2018
30. Institute JB. Critical Appraisal Tools [Available from: http://joannabriggs.org/research/critical-appraisal-tools.html accessed 5 august 2018.
31. Weingarten MA, Paul M, Leibovici L. Assessing ethics of trials in systematic reviews. *Bmj* 2004;328(7446):1013-14.
32. Ouzzani M, Hammady H, Fedorowicz Z, et al. Rayyan—a web and mobile app for systematic reviews. *Systematic reviews* 2016;5(1):210.
33. Braun V, Clarke V. Using thematic analysis in psychology. *Qualitative research in psychology* 2006;3(2):77-101.
34. Maguire M, Delahunt B. Doing a thematic analysis: A practical, step-by-step guide for learning and teaching scholars. *AISHE-J: The All Ireland Journal of Teaching and Learning in Higher Education* 2017;9(3)
35. Cushing A, Abbott S, Lothian D, et al. Peer feedback as an aid to learning - What do we want? Feedback. When do we want it? Now! *Medical Teacher* 2011;33(2) doi: 10.3109/0142159X.2011.542522
36. Iqbal M, Velan GM, O'Sullivan AJ, et al. Differential impact of student behaviours on group interaction and collaborative learning: medical students' and tutors' perspectives. *BMC medical education* 2016;16(1):217-17. doi: 10.1186/s12909-016-0730-1
37. Nofziger AC, Naumburg EH, Davis BJ, et al. Impact of peer assessment on the professional development of medical students: a qualitative study. *Academic medicine : journal of the Association of American Medical Colleges* 2010;85(1):140-47. doi: 10.1097/ACM.0b013e3181c47a5b
38. Emke AR, Cheng S, Chen L, et al. A Novel Approach to Assessing Professionalism in Preclinical Medical Students Using Multisource Feedback Through Paired Self- and Peer Evaluations. *Teaching and Learning in Medicine* 2017;29(4):402-10. doi: 10.1080/10401334.2017.1306446
39. Chou CL, Masters DE, Chang A, et al. Effects of longitudinal small-group learning on delivery and receipt of communication skills feedback. *Medical Education* 2013;47(11):1073-79. doi: 10.1111/medu.12246
40. Tai JH-M, Canny BJ, Haines TP, et al. The role of peer-assisted learning in building evaluative judgement: opportunities in clinical medical education. *Advances in health sciences education : theory and practice* 2016;21(3):659-76. doi: 10.1007/s10459-015-9659-0
41. Cho Y, Je S, Yoon YS, et al. The effect of peer-group size on the delivery of feedback in basic life support refresher training: A cluster randomized controlled trial. *BMC Medical Education* 2016;16(1):1-8. doi: 10.1186/s12909-016-0682-5

For peer review only

For peer review only - http://bmjopen.bmj.com/site/about/guidelines.xhtml

42. Moineau G, Power B, Pion A-MJ, et al. Comparison of student examiner to faculty examiner scoring and feedback in an OSCE. *Medical education* 2011;45(2):183-91. doi: 10.1111/j.1365-2923.2010.03800.x
43. Satterthwaite JD, Grey NJA. Peer-group assessment of pre-clinical operative skills in restorative dentistry and comparison with experienced assessors. *European Journal of Dental Education* 2008;12(2):99-102. doi: 10.1111/j.1600-0579.2008.00509.x
44. Vaughn CJ, Kim E, O'Sullivan P, et al. Peer video review and feedback improve performance in basic surgical skills. *American Journal of Surgery* 2016;211(2):355-60. doi: 10.1016/j.amjsurg.2015.08.034
45. Tricio J, Woolford M, Escudier M. Analysis of dental students' written peer feedback from a prospective peer assessment protocol. *European Journal of Dental Education* 2016;20(4):241-47.
46. Rees C, Sheard C, McPherson A. Communication skills assessment: the perceptions of medical students at the University of Nottingham. *Medical Education* 2002;36(9):868-78.
47. Spandorfer J, Puklus T, Rose V, et al. Peer assessment among first year medical students in anatomy. *Anatomical sciences education* 2014;7(2):144-52. doi: 10.1002/ase.1394
48. Martin D, Friesen E, De Pau A. Three heads are better than one: a mixed methods study examining collaborative versus traditional test-taking with nursing students. *Nurse education today* 2014;34(6):971-77. doi: 10.1016/j.nedt.2014.01.004
49. Mui Lim S, Rodger S. The use of interactive formative assessments with first-year occupational therapy students. *International Journal of Therapy and Rehabilitation* 2010;17(11):576-86.
50. Elshami W, Abdalla ME. Diagnostic radiography students' perceptions of formative peer assessment within a radiographic technique module. *Radiography (London, England : 1995)* 2017;23(1):9-13. doi: 10.1016/j.radi.2016.06.001
51. Koh LC. Academic staff perspectives of formative assessment in nurse education. *Nurse education in practice* 2010;10(4):205-09. doi: 10.1016/j.nepr.2009.08.007
52. Arnold L, Shue CK, Kritt B, et al. Medical students' views on peer assessment of professionalism. *Journal of General Internal Medicine* 2005;20(9):819-24. doi: 10.1111/j.1525-1497.2005.0162.x
53. Carlson E, Stenberg M, Chan B, et al. Nursing as universal and recognisable: Nursing students' perceptions of learning outcomes from intercultural peer learning webinars: A qualitative study. *Nurse education today* 2017;57:54-59.
54. Homberg A, Hundertmark J, Krause J, et al. Promoting medical competencies through a didactic tutor qualification programme–a qualitative study based on the CanMEDS Physician Competency Framework. *BMC Medical Education* 2019;19(1):1-8.
55. Schwill S, Fahrbach-Veeser J, Moeltner A, et al. Peers as OSCE assessors for junior medical students–a review of routine use: a mixed methods study. *BMC medical education* 2020;20(1):1-12.
56. Vickerman P. Student perspectives on formative peer assessment : an attempt to deepen learning ? 2009;34(2):221-30. doi: 10.1080/02602930801955986
57. Sluijsmans D, Prins F. A conceptual framework for integrating peer assessment in teacher education. *Studies in Educational Evaluation* 2006;32(1):6-22.
58. Boud D, Falchikov N. Developing assessment for informing judgement. *Rethinking assessment in higher education: Learning for the longer term* 2007:181-97.
59. Cassidy S. Developing employability skills : peer assessment in higher education. 2006 doi: 10.1108/00400910610705890
60. Solheim E, Plathe HS, Eide H. Nursing students' evaluation of a new feedback and reflection tool for use in high-fidelity simulation – Formative assessment of clinical skills. A descriptive quantitative research design. *Nurse Education in Practice* 2017;27:114-20. doi: 10.1016/j.nepr.2017.08.021
61. Topping KJ. Peer assessment. *Theory into practice* 2009;48(1):20-27.

For peer review only - http://bmjopen.bmj.com/site/about/guidelines.xhtml

62. Casey D, Burke E, Houghton C, et al. Use of peer assessment as a student engagement strategy in nurse education. *Nursing and Health Sciences* 2011;13(4):514-20. doi: 10.1111/j.1442-2018.2011.00637.x
63. Papinczak T, Young L, Groves M, et al. An analysis of peer, self, and tutor assessment in problem-based learning tutorials. *Med Teach* 2007;29(5):e122-32. doi: 10.1080/01421590701294323 [published Online First: 2007/09/22]
64. Kollar I, Fischer F. Peer assessment as collaborative learning: A cognitive perspective. *Learning and Instruction* 2010;20(4):344-48.
65. Finn GM, Garner J. Twelve tips for implementing a successful peer assessment. *Medical teacher* 2011;33(6):443-46.
66. Jackel B, Pearce J, Radloff A, et al. Assessment and feedback in higher education.
67. Gentles SJ, Lokker C, McKibbon KA. Health information technology to facilitate communication involving health care providers, caregivers, and pediatric patients: a scoping review. *Journal of medical Internet research* 2010;12(2):e22.
68. Pham MT, Rajić A, Greig JD, et al. A scoping review of scoping reviews: advancing the approach and enhancing the consistency. *Research synthesis methods* 2014;5(4):371-85.

For peer review only

For peer review only - http://bmjopen.bmj.com/site/about/guidelines.xhtml

Table 1. The PCC mnemonic as recommended by the Joanna Briggs Institute

Population	Concept	Context
Students assessing students	Intervention, rationale, outcome, context, and students' and teachers' experience of formative peer assessment	Healthcare education programs in higher education

For peer review only

For peer review only - http://bmjopen.bmj.com/site/about/guidelines.xhtml

Table 2. Overview of included studies.

Author, year, country, and journal	Aim	Design	Participants	Main findings	Quality*
Arnold et al., 2005,[52] USA<br><br>*Journal of General Internal Medicine*	Identify factors that encourage or discourage student participation in peer assessment	Qualitative<br>Grounded theory<br><br>Focus groups (16) at two medical schools	n=61, medical students in year 1, 3, and 4	The characteristics of the peer assessment system and the environment can encourage or discourage participation. Themes: (1) Students' struggle with peer assessment, (2) Characteristics of a peer assessment system, and (3) Environmental factors	moderate
Cho et al., 2016,[41] England<br><br>*BMC Medical Education*	Investigate the effect of peer-group size on competency-based skills	Quantitative<br>Cluster RCT	n=115, medical students in year 6	Smaller groups (4.1) show more active and preferred than large groups (8,1). Group size did not impact scores.	high
Chou et al., 2013,[39] USA<br><br>*Medical Education*	Examine the role of prior peer-learning relationships between students in their delivery and receipt of feedback on clinical communication skills	Mixed method<br>Case-control<br>Descriptive statistics<br>Survey, video observations	n= 72 medical students in year 3 with prior peer learning relationships<br><br>n=36 students in control group with no prior peer relationships.	Students with prior peer learning relationships more likely to provide specific corrective feedback than those without prior relationships. No significant difference between groups regarding how feedback was received.	moderate
Cushing et al., 2011,[35] United Kingdom<br><br>*Medical Teacher*	Investigate the benefits of formative peer feedback in communication skills and develop a training programme in peer feedback	Mixed method<br>Questionnaire (20 items) at two occasions with 6 months in between.<br>Focus groups (5 medical- and 2 nurse students)	n=45 medical students in year 1<br><br>n=48 nursing students in year 1	Students valued the learning opportunity of both being examiner and observer. They preferred more in-depth feedback and feedback from tutors. They expressed anxiety about giving negative feedback to a peer and had mixed views on giving feedback (relaxed or pressured) and its use in clinical placements.	high
Elshami & Abdalla, 2017,[50]<br>United Arab Emirates<br><br>*Radiography*	Assess perception of formative peer assessment	Qualitative<br>Action research<br>Focus groups (3)<br>Content analysis	n=19 (24**) diagnostic radiography students in year 3	Formative peer assessment gives valuable feedback from same level or more experienced peers. Need for training and detailed rubrics.	moderate
Emke et al., 2017,[38] USA	Demonstrate that perceptual errors related to professionalism behaviors can be detected early through	Quantitative	n=246 medical students in year 2	Multiple peer assessments and feedback a tool predictor of unprofessional behavior.	moderate

For peer review only - http://bmjopen.bmj.com/site/about/guidelines.xhtml

Teaching and Learning in Medicine	repeated multisource feedback				
Iqbal et al., 2016,[36]<br>Australia<br><br>*BMC Medical Education*	Explore students' and tutors' perception of key collaborative behaviors that impact collaborative learning and interaction	Qualitative<br>Focus groups (5) with students<br>Interviews (8) with teachers<br>Thematic analysis	n= 22 medical students in year one and two<br><br>n= 8 teachers	Being respectful, giving constructive feedback, and being engaged and prepared had positive impact on both learning and group interaction. Passiveness, unreliability, irresponsibility, and condescending attitudes had a negative impact on learning and interaction. Similar results from teachers.	high
Koh, 2010,[51]<br>United Kingdom<br><br>*Nurse Education in Practice*	Explore how academic staff experience, understand, and interpret the process of formative assessment and feedback of theoretical assessment	Qualitative<br>Phenomenology<br>Semi-structured interviews (22)<br>Thematic analysis	n=20 academic staff in nurse education	Teachers see themselves as key facilitators and think students prefer teacher feedback. Students are assumed to have the skill to peer assess and give feedback but are unprepared and need support and introduction early in education. Teachers need professional development themselves.	moderate
Mui Lim & Rodger, 2010.[49]<br>Australia<br><br>*International Journal of Therapy and Rehabilitation*	Improve students learning through interactive formative assessment and student generated questions	Mixed methods<br>Cohort study<br>Evaluation questionnaire	n= 115 occupational therapy students in year 1 in 2009 compared with n= 98 students in 2008	Significant improvement in exams result from being part of interactive formative assessment, which is beneficial for learning and identifying knowledge gaps.	moderate
Martin, Friesen, & De Pau, 2014,[48]<br>Canada<br><br>*Nurse Education Today*	Examine collaborative testing versus traditional test taking with undergraduate nursing students in a nine-station OSCE	Mixed method<br>Cross over design<br>Survey<br>Focus groups	n=70 nursing students	Significantly higher scores in collaborative testing than in traditional testing.<br>Themes: (1) studying more/studying differently, (2)/ cognitive collectivism (3), "it stuck in my head better" (4), confidence, and (5) practicing how to share knowledge and negotiate.	moderate
Moineau et al., 2011,[42]<br>Canada<br><br>*Medical Education*	Compare scores and experiences of formative assessment from faculty and senior students during OSCE-examinations	Quantitative<br>Cross sectional<br>Pre- and post-questionnaire	n=66 medical students in year 2<br>n=27 year 4 student examiners<br>n=27 teaching doctors	Students (year 4) assessing students (year 2) with checklists in OSCE-examinations equally assessed compared to faculty members. A positive learning experience expressed from both students and faculty.	moderate
Nofziger et al., 2010,[37]	Investigate the impact of peer assessment on future	Qualitative	n=70 medical students in year 2	67% found peer assessment helpful, reassuring, or confirming something they	moderate

For peer review only - http://bmjopen.bmj.com/site/about/guidelines.xhtml

USA<br><br>*Academic Medicine*	professional development and students' experiences	Questionnaire and narrative comments<br>Frequency count	n=48 in year 4	knew; 65% reported important transformations in awareness, attitudes, or behaviors because of peer assessment. Change was more likely when feedback was specific and described an area for improvement.	
Rees, Sheard, & McPherson, 2002,[46]<br>United Kingdom<br><br>*Medical Education*	Explore students' perceptions of communication skill assessment	Qualitative<br>Focus groups	n=7 medical students in year 1<br>n= 7 in year 2<br>n=10 in year 3<br>n= 5 in year 4<br>n=3 in year 5	Year 4 and 5 more positive than younger students. Opportunities to compare communication skills with peers from same level. Learning experience being the assessor. No constructive criticism from peers. Difficult to be objective and to give feedback.	high
Satterthwaite & Grey, 2008,[43]<br>United Kingdom<br><br>*European Journal of Dental Education*	Investigate if any differences existed between marks given by a peer group and those given by experienced assessors	Quantitative<br>Cross sectional	n=65 dental students	No significant difference in grades between experienced examiners and peer group.	moderate
Spandorfer et al., 2014,[47]<br>USA<br><br>*Anatomical Science Education*	Determine whether peer assessment improves students work habits and interpersonal attributes and whether it is accepted by students, focusing on low performing students	Multi-methods<br>Paired sample t-test<br>Pearson correlation coefficients<br>Survey- content analysis	n=267 medical students in year 1; follow-up in year 2	Significant improvement after on-line peer feedback between test 1 and 2.<br>Themes: (1) Initiative, (2) Communication, (3) Respect, (4) Preparation, and (5) Focus. Students prefer anonymous feedback from peers.	moderate
Tai et al., 2016,[40]<br>Australia<br><br>*Advances in Health Science Education*	Investigate students' experience of peer-assisted learning.	Mixed methods<br>Ethnographic<br>Survey, observations, and interviews<br>Thematic analysis	n=10 medical students in year 1 (observed)<br><br>n=191 students in year 3 (survey)	Observing and giving feedback to peers contributed to learning, but students value feedback from teachers for validation. Students want to preserve social relationships with peers; therefore, feedback is not so constructive. Peers provide a supportive learning environment.	high
Tricio, Woolford, & Escudier, 2016,[45]<br>United Kingdom<br><br>*European Journal of Dental Education*	Analyze written feedback provided as a part of a formative and structured peer assessment protocol.	Multi-methods<br>Descriptive statistic<br>Thematic analysis	n=40 dental students in year 2 in pre-clinical skills laboratory<br><br>n=68 dental students in year 5 in clinic	Year 2 focuses on practical and clinical knowledge; in contrast, year 5 focuses comments on communication, management, and leadership. Year 2 gives more positive comments on peer performance than year 5.	moderate

For peer review only - http://bmjopen.bmj.com/site/about/guidelines.xhtml

Vaughn et al., 2016,[44] USA<br><br>*The American Journal of Surgery*	Evaluate the use, quality, and quantity of peer video feedback and compare peers and faculty feedback.	Quantitative<br>Cross sectional<br>Paired t-test, Mann-Whitney statistic<br>Survey	n=24 medical students***	Significant change in performance across 3 periods in both groups. Peer feedback group performed better at final assessment than faculty feedback group (not significant). Peers gave higher scores than faculty. No significant differences when using a checklist.	moderate

*High equals majority of items in the critical appraisal tools.

**24 students included in the intervention, and 19 attended the focus group session.

***12 students received faculty feedback, and 12 students received peer feedback.

For peer review only

Table 3. Overview and summery of the context, rationale, and interventions of formative peer assessment presented in the included studies.

Contexts	Rationales	Interventions
Intra-professional students (17)*<br>Combination of medical and nursing students (1)<br><br><u>Conducted in the following:</u><br>Clinical skill labs (11)<br>Theoretical courses (7)<br>Combination of theoretical and clinical placement course (1)<br><br>Within an educational model as problem-based learning, peer learning, or peer assisted learning (7)	<u>Giving and receiving feeback supports student learning:</u><br>Promotes learning (8)<br>Enhances critical thinking (1)<br>Promotes understanding of the assessment process (1)<br>Develops critical- and interpersonal skills (1)<br>Helps identify knowledge gaps (1)<br>Supports low-performance students (1)<br><br><u>It prepares students for knowledge-related professionalism in the healthcare profession by helping them identify the following:</u><br>Professional- and un-professional behavior (6)<br>Clinical competence (2)<br>Technical skills (2)<br>Communication skills (2)<br>Collaborative behavior (2)<br>Evaluative judgement (1)<br><br><u>It enhances teachers' teaching (1)</u><br><br><u>It provides cost benefits:</u><br>Students as assessors instead of teachers (2)<br>Students as creators of the learning activities instead of teachers (1)	<u>Introduction (in workshops):</u><br>Preparations in giving and receiving feedback (3)<br>Introduction of guidelines or checklists to guide the peer assessor (3)<br>Introduction of the learning activity (2)<br>Preparation in communication (1)<br><br><u>Learning activities focusing feedback on professionalism:</u><br>Clinical skills (3)<br>Collaborative behavior (2)<br>Clinical reasoning (2)<br>Theoretical knowledge (2)<br>Communication skills (2)<br>Management skills (1)<br><br><u>Feedback types:</u><br>Face-to-face (7)<br>Anonymous (5)<br>Written (3) or through observations (3)<br>Interactive on-line assessment (3)<br>Grading of the given feedback (1)<br><br>Random peers (8)<br>Ability to choose peer (1)<br>In small groups < 6 (6)<br>In large groups > 6 (3)

* = appears in how many of the included 18 studies

For peer review only

For peer review only

For peer review only - http://bmjopen.bmj.com/site/about/guidelines.xhtml

Figure 1: PRISMA Flow Diagram

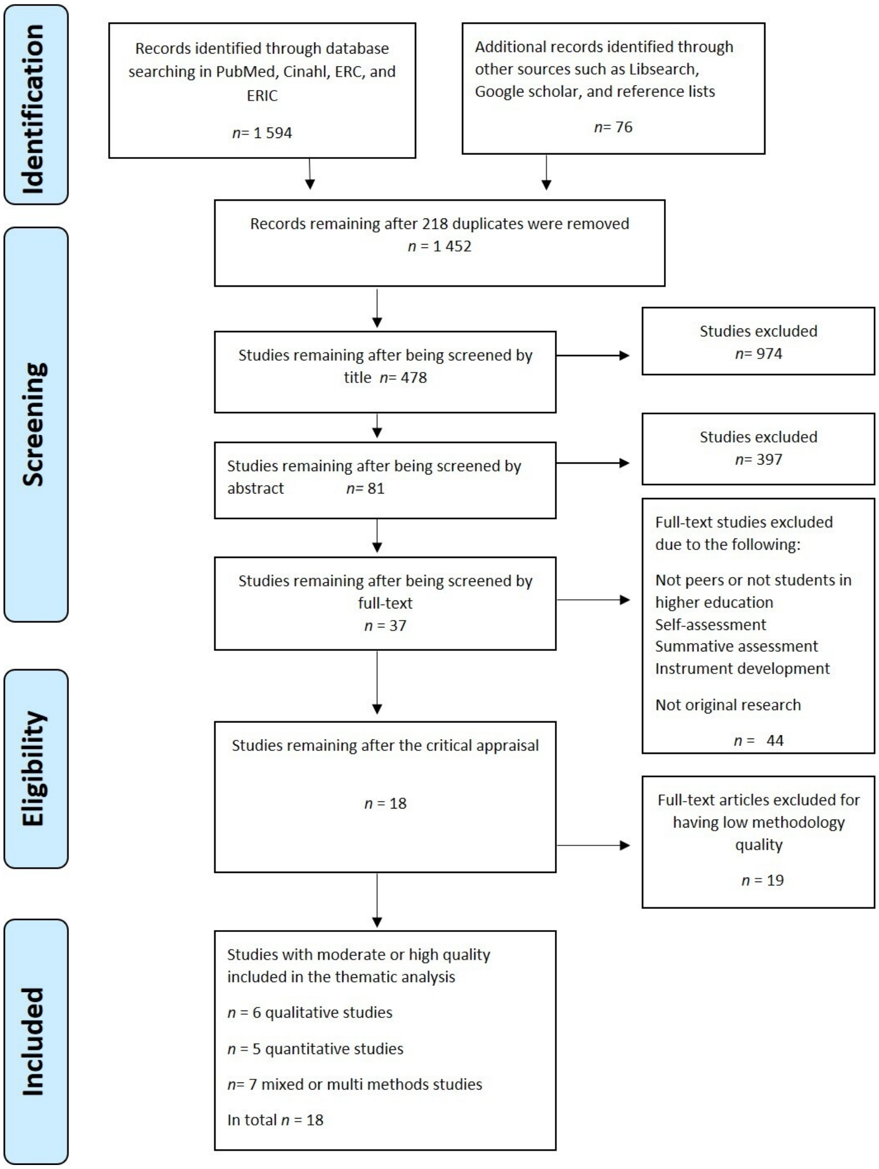

100x122mm (300 x 300 DPI)

Appendix 1. Presentation of the search strategy and results from the Pub Med database until 28th of May, 2019.

	Search block 1: Healthcare education
#	MESH-terms
1	"Students, Medical"[Mesh] OR
2	"Students, Nursing"[Mesh] OR
3	"Students, Dental"[Mesh] OR
4	"Students, Health Occupations"[Mesh] OR
5	"Education, Medical"[Mesh] OR
6	"Education, Nursing"[Mesh] OR
7	"Education, Dental" [Mesh] OR
8	"Midwifery/education"[Mesh] OR
9	"Allied Health Personnel/education"[Mesh]
10	S1 OR S2 OR S3 OR S4 OR S5 OR S6 OR S7 OR S8 OR S9
#	Free text terms
11	"medical student*" OR
12	"nursing student*" OR
13	"midwifery student* OR
14	"dental student*" OR
15	"physical therapy student*" OR
16	"occupational therapy student*" OR
17	"allied health student*" OR
18	"health occupations student*" OR
19	"health care stud*" OR
20	"Health care education" OR
21	"health science education" OR
22	"Medical education" OR
23	" Nursing education" OR
24	"Dental education" OR
25	"allied health education" OR
26	"Health occupation* education*" OR
27	"midwifery education"
28	S11 OR S12 OR S13 OR S14 ORS15 OR S16 OR S17 OR S18 OR S19 OR S20 OR S21 OR S22 OR S23 OR S24 OR S25 OR S26 OR S27
29	S10 OR S28

	Search block 2: Peer assessment
#	MESH
31	"Educational Measurement"[Mesh] OR
32	"Peer Group"[Mesh] OR
33	"Peer Review"[Mesh]
34	S31 OR S32 OR S33
#	Free text terms
35	"peer assessment" OR
36	"peer evaluation" OR
37	"peer observation" OR
38	"peer feedback" OR
39	"peer review" OR
40	"peer assess*" OR
41	"*peer assess" OR
42	"peer examiner" OR
43	"peer grad*" OR
44	"peer group" OR
45	"Student performance appraisal" OR
46	"educational measurement"
47	S35 OR S36 OR S37 OR S38 OR S39 OR S40 OR S41 OR S42 OR S43 OR S44 OR S45 OR S46
48	S34 OR S47

For peer review only

	Search block 3: Formative assessment
#	MESH
49	"Formative Feedback"[Mesh]
#	Free text terms
50	"Formative evaluation" OR
51	"Formative feedback" OR
52	"Formative assessment" OR
53	"Formative* assess*" OR
54	"Formativ* evaluation" OR
55	Formativ* OR
56	"formative evaluation research"
57	S37 OR S38 OR S39 OR S40 OR S41 OR S42 OR S43
58	S49 OR S57

#	Combination of search blocks 1, 2, and 3
59	S29 AND S48 AND S58

For peer review only

Appendix 2. Charting form

CHARTING FORM

ARTICLE NO:

TITLE:

AUTHOR/S:

YEAR OF PUBLICATION:

COUNTRY:

RESEARCH DESIGN: QUANTITATIVE ☐ QUALITATIVE ☐ OTHER: ☐ ______

METHOD:

AIM:

NUMBER OF PARTICIPANTS (n=):

SETTING:

MEDICAL EDUCATION ☐

NURSING EDUCATION ☐

MIDWIFERY EDUCATION ☐

PHYSIOTHERAPY EDUCATION ☐

OCCUPATIONAL THERAPY ☐

DENTAL EDUCATION ☐

OTHER ☐ ______

For peer review only

DOES THE STUDY PRESENT?

HOW PEER ASSESSEMENT INTERVENTIONS ARE DELIVERED	YES	NO	
PEER ASSESSMENT	YES	NO	
FORMATIVE ASSESSMENT	YES	NO	
STUDENTS EXPERIENCE	YES	NO	
TEACHERS EXPERIENCE	YES	NO	OTHER:
OUTCOME/S OF PEER ASSESSMENT INTERVENTION	YES	NO	
RATIONALE/S FOR PEER ASSESSMENT INTERVENTION	YES	NO	

MAIN FINDINGS:

INCLUDED: YES NO

REASON/S FOR EXCLUSION:

For peer review only

(Only to be answered if the study is to be included)

Grading according to the Critical Appraisal Skills Program (CASP)

QUALITATIVE STUDIES AND SYSTEMATIC REVIEWS

LOW MODERATE HIGH

RCT AND CASE CONTROL STUDIES

LOW MODERATE HIGH

COHORT STUDIES

LOW MODERATE HIGH

Grading according to The Mixed Method Appraisal Tool

MIXED METHOD STUDIES

LOW MODERATE HIGH

Grading according to the Joanna Briggs Institute Critical Appraisal Tool

QUASI-EXPERIMENTAL STUDIES

LOW MODERATE HIGH

ANALYTICAL CROSS SECTIONAL STUDIES

LOW MODERATE HIGH

CASE REPORTS

LOW MODERATE HIGH

Reviewed by:

(Signature and date)

For peer review only

Preferred Reporting Items for Systematic reviews and Meta-Analyses extension for Scoping Reviews (PRISMA-ScR) Checklist

SECTION	ITEM	PRISMA-ScR CHECKLIST ITEM	REPORTED ON PAGE #
**TITLE**			
Title	1	Identify the report as a scoping review.	1
**ABSTRACT**			
Structured summary	2	Provide a structured summary that includes (as applicable): background, objectives, eligibility criteria, sources of evidence, charting methods, results, and conclusions that relate to the review questions and objectives.	2
**INTRODUCTION**			
Rationale	3	Describe the rationale for the review in the context of what is already known. Explain why the review questions/objectives lend themselves to a scoping review approach.	3, 4
Objectives	4	Provide an explicit statement of the questions and objectives being addressed with reference to their key elements (e.g., population or participants, concepts, and context) or other relevant key elements used to conceptualize the review questions and/or objectives.	5
**METHODS**			
Protocol and registration	5	Indicate whether a review protocol exists; state if and where it can be accessed (e.g., a Web address); and if available, provide registration information, including the registration number.	4,13
Eligibility criteria	6	Specify characteristics of the sources of evidence used as eligibility criteria (e.g., years considered, language, and publication status), and provide a rationale.	5, Table 1
Information sources*	7	Describe all information sources in the search (e.g., databases with dates of coverage and contact with authors to identify additional sources), as well as the date the most recent search was executed.	6, Appendix 1
Search	8	Present the full electronic search strategy for at least 1 database, including any limits used, such that it could be repeated.	Appendix 1
Selection of sources of evidence†	9	State the process for selecting sources of evidence (i.e., screening and eligibility) included in the scoping review.	6,7
Data charting process‡	10	Describe the methods of charting data from the included sources of evidence (e.g., calibrated forms or forms that have been tested by the team before their use, and whether data charting was done independently or in duplicate) and any processes for obtaining and confirming data from investigators.	6,7, Appendix 2
Data items	11	List and define all variables for which data were sought and any assumptions and simplifications made.	5, Appendix 1
Critical appraisal of individual sources of evidence§	12	If done, provide a rationale for conducting a critical appraisal of included sources of evidence; describe the methods used and how this information was used in any data synthesis (if appropriate).	6, 7

St. Michael's
Inspired Care.
Inspiring Science.

For peer review only - http://bmjopen.bmj.com/site/about/guidelines.xhtml

SECTION	ITEM	PRISMA-ScR CHECKLIST ITEM	REPORTED ON PAGE #
Synthesis of results	13	Describe the methods of handling and summarizing the data that were charted.	6,7
**RESULTS**			
Selection of sources of evidence	14	Give numbers of sources of evidence screened, assessed for eligibility, and included in the review, with reasons for exclusions at each stage, ideally using a flow diagram.	5,6, Figure 1
Characteristics of sources of evidence	15	For each source of evidence, present characteristics for which data were charted and provide the citations.	7, Table 2
Critical appraisal within sources of evidence	16	If done, present data on critical appraisal of included sources of evidence (see item 12).	Table 2
Results of individual sources of evidence	17	For each included source of evidence, present the relevant data that were charted that relate to the review questions and objectives.	7,8,9,10, Table 2
Synthesis of results	18	Summarize and/or present the charting results as they relate to the review questions and objectives.	8,9,10, Table 3
**DISCUSSION**			
Summary of evidence	19	Summarize the main results (including an overview of concepts, themes, and types of evidence available), link to the review questions and objectives, and consider the relevance to key groups.	10, 11
Limitations	20	Discuss the limitations of the scoping review process.	12
Conclusions	21	Provide a general interpretation of the results with respect to the review questions and objectives, as well as potential implications and/or next steps.	11, 12
**FUNDING**			
Funding	22	Describe sources of funding for the included sources of evidence, as well as sources of funding for the scoping review. Describe the role of the funders of the scoping review.	12

JBI = Joanna Briggs Institute; PRISMA-ScR = Preferred Reporting Items for Systematic reviews and Meta-Analyses extension for Scoping Reviews.

* Where *sources of evidence* (see second footnote) are compiled from, such as bibliographic databases, social media platforms, and Web sites.

† A more inclusive/heterogeneous term used to account for the different types of evidence or data sources (e.g., quantitative and/or qualitative research, expert opinion, and policy documents) that may be eligible in a scoping review as opposed to only studies. This is not to be confused with *information sources* (see first footnote).

‡ The frameworks by Arksey and O'Malley (6) and Levac and colleagues (7) and the JBI guidance (4, 5) refer to the process of data extraction in a scoping review as data charting.

§ The process of systematically examining research evidence to assess its validity, results, and relevance before using it to inform a decision. This term is used for items 12 and 19 instead of "risk of bias" (which is more applicable to systematic reviews of interventions) to include and acknowledge the various sources of evidence that may be used in a scoping review (e.g., quantitative and/or qualitative research, expert opinion, and policy document).

From: Tricco AC, Lillie E, Zarin W, O'Brien KK, Colquhoun H, Levac D, et al. PRISMA Extension for Scoping Reviews (PRISMA-ScR): Checklist and Explanation. Ann Intern Med. ;169:467–473. doi: 10.7326/M18-0850

For peer review only - http://bmjopen.bmj.com/site/about/guidelines.xhtml
